# Parallel metatranscriptome analyses of host and symbiont gene expression in the gut of the termite *Reticulitermes flavipes*

**DOI:** 10.1186/1754-6834-2-25

**Published:** 2009-10-15

**Authors:** Aurélien Tartar, Marsha M Wheeler, Xuguo Zhou, Monique R Coy, Drion G Boucias, Michael E Scharf

**Affiliations:** 1 Department of Entomology and Nematology, University of Florida, Gainesville, FL, USA; 2 Division of Math, Science and Technology, Nova Southeastern University, Fort Lauderdale, FL, USA; 3 Current address : Department of Entomology, University of Illinois, Champaign-Urbana, IL, USA; 4 Current address : Department of Entomology, University of Kentucky, Lexington, KY, USA

## Abstract

**Background:**

Termite lignocellulose digestion is achieved through a collaboration of host plus prokaryotic and eukaryotic symbionts. In the present work, we took a combined host and symbiont metatranscriptomic approach for investigating the digestive contributions of host and symbiont in the lower termite *Reticulitermes flavipes*. Our approach consisted of parallel high-throughput sequencing from (i) a host gut cDNA library and (ii) a hindgut symbiont cDNA library. Subsequently, we undertook functional analyses of newly identified phenoloxidases with potential importance as pretreatment enzymes in industrial lignocellulose processing.

**Results:**

Over 10,000 expressed sequence tags (ESTs) were sequenced from the 2 libraries that aligned into 6,555 putative transcripts, including 171 putative lignocellulase genes. Sequence analyses provided insights in two areas. First, a non-overlapping complement of host and symbiont (prokaryotic plus protist) glycohydrolase gene families known to participate in cellulose, hemicellulose, alpha carbohydrate, and chitin degradation were identified. Of these, cellulases are contributed by host plus symbiont genomes, whereas hemicellulases are contributed exclusively by symbiont genomes. Second, a diverse complement of previously unknown genes that encode proteins with homology to lignase, antioxidant, and detoxification enzymes were identified exclusively from the host library (laccase, catalase, peroxidase, superoxide dismutase, carboxylesterase, cytochrome P450). Subsequently, functional analyses of phenoloxidase activity provided results that were strongly consistent with patterns of laccase gene expression. In particular, phenoloxidase activity and laccase gene expression are mostly restricted to symbiont-free foregut plus salivary gland tissues, and phenoloxidase activity is inducible by lignin feeding.

**Conclusion:**

To our knowledge, this is the first time that a dual host-symbiont transcriptome sequencing effort has been conducted in a single termite species. This sequence database represents an important new genomic resource for use in further studies of collaborative host-symbiont termite digestion, as well as development of coevolved host and symbiont-derived biocatalysts for use in industrial biomass-to-bioethanol applications. Additionally, this study demonstrates that: (i) phenoloxidase activities are prominent in the *R. flavipes *gut and are not symbiont derived, (ii) expands the known number of host and symbiont glycosyl hydrolase families in *Reticulitermes*, and (iii) supports previous models of lignin degradation and host-symbiont collaboration in cellulose/hemicellulose digestion in the termite gut. All sequences in this paper are available publicly with the accession numbers FL634956-FL640828 (Termite Gut library) and FL641015-FL645753 (Symbiont library).

## Background

Lignocellulose, the principal constituent of plant biomass, is currently targeted as a primary feedstock for the production of biofuels [1]. However, the extant industrial processes used for fuel conversion suffers from a lack of efficiency that has been related in part to the extreme recalcitrance of plant-produced lignocellulose [2,3]. Biofuel production from plants requires the successful depolymerization of the large carbohydrate polymers cellulose and hemicellulose. In lignocellulose these polymers are not directly accessible for chemical or enzymatic treatments but are combined with lignins and other molecules to form a complex, and highly resistant, three-dimensional molecular structure. At present, the inability to efficiently extract simple, utilizable sugars from lignocellulose through depolymerization reactions is a significant limiting factor for the bioethanol industry [1]. One strategy for identification of novel enzymes to improve this process is through the mining of genomes of lignocellulolytic organisms, both prokaryotic and eukaryotic [4,5].

Termites (Insecta, Isoptera) are ubiquitous arthropods that efficiently digest lignocellulose and flourish on this seemingly nutritionally poor diet. The feeding activity of termites, combined with their preference for wood, is known to play a critical role in ecosystem nutrient recycling, and to cause significant economic damage to human-built structures. Recently, the ability of termites to convert recalcitrant plant biomass into a useable energy source has attracted much interest due to the numerous potential applications in biofuel production [6-8]. Lignocellulose digestion in termites is intimately correlated with the presence of a highly specific flora of symbiotic microbes [9,10]. Many of these microbes, essential for the survival of the termite hosts, reside in a modified hindgut or fermentation sac [11,12].

Current termite lignocellulose digestion models consider both host and symbiont inputs (for example, [5,13,14]). In this respect, recent studies conducted on the lower termites *Coptotermes formosanus *and *Reticulitermes flavipes *have demonstrated that carbohydrate-active enzymes of both termite and symbiont origins are produced simultaneously but in different regions of the gut [15-18]. For example, termites and symbiotic protists produce glycosyl hydrolases that belong to two separate families (GHFs) (respectively): GHF9 endoglucanases and GHF7 exoglucanases/cellobiohydrolases [16,17]. Aside from semantic disagreements [19], there is general agreement that sequential depolymerization reactions involve insect and symbiont enzymes with different and possibly complementary properties, allowing for efficient lignocellulose digestion [6,16,17]. The respective contributions and requirements of each enzyme, as well as the role played by cellulases produced by prokaryotic symbionts, have yet to be fully understood.

In efforts to define termite lignocellulolytic processes, several previous studies have completed metagenome, transcriptome, or metatranscriptome sequencing projects on various termite digestive organs or symbiotic gut fauna. Two studies have focused specifically on symbionts sampled from the hindgut lumen. First, a metatranscriptomic project to sequence cDNA clones representing expressed genes of eukaryotic (protistan) symbionts from the lower *Reticulitermes speratus *was reported by Todaka *et al*. [20]. This study produced 910 expressed sequence tag (EST) sequences that aligned into 580 tentative genes. The *R. speratus *study revealed many diverse cellulase and hemicellulase sequences, in particular, an unexpectedly large complement of GHF7 cellulases putatively involved in microcrystalline cellulose degradation. Second, a metagenomic project to sequence prokaryotic gut symbiont genomes of a higher *Nasutitermes *termite (species unknown) produced over 100 million bases of DNA sequence [21] (see also [7,8,22]). This study revealed genes containing more than 700 unique glycoside hydrolase catalytic domains from 45 different carbohydrate active gene families, as well as other genes relating to numerous aspects of microbial life in the gut microenvironment, such as nitrogen fixation and H_2 _production. While both of these first two studies identified rich complements of cellulases, hemicellulases, and other carbohydrolases, they revealed no evidence of lignases.

Still other large-scale sequencing projects have focused on endosymbionts and ectosymbionts; specifically, bacterial endosymbionts of protist gut symbionts and a termite-cultivated symbiotic fungus. Two studies sequenced the entire genomes of bacterial endosymbionts of hindgut protists [23,24]. These bacteria included 'RS-D17', a bacterium from the protist gut symbiont *Trichonympha agilis *of the termite *R. speratus*; and 'CfPt1-2', a Bacteroidales endosymbiont of the protist gut symbiont *Pseudotrichonympha grassii *of the termite *C. formosanus*. Both of these bacterial phylotypes are as yet unculturable and occur only within cellulolytic protists of termite guts. Interestingly, genome sequencing revealed that these bacteria are highly adept at nitrogen fixation, monosaccharide metabolism and amino acid production, but encode no lignocellulase genes and thus, likely play no roles in lignocellulose degradation. The termite fungal cultivar work was performed by sequencing cDNA ESTs from *Termitomyces*, a fungal ectosymbiont obtained from mounds of the higher termite *Macrotermes gilvus *[25]. This work identified 1,582 tentatively unique gene sequences including well represented pectinases, hemicellulases and cellulases, as well as 2 laccases putatively involved in lignin degradation. Thus, while bacterial endosymbionts of protists confer no lignocellulose digestion capabilities, cultivated fungal symbionts found in some higher termite nests clearly do.

As exemplified by the above-summarized work, termite gut symbionts play well established roles in cellulose and hemicellulose digestion; however, there is no sequence-based evidence to date supporting that gut symbionts are capable of lignin degradation. Furthermore, it is also well established that both lower and higher termites actively secrete endogenous cellulases that play important roles in cellulose digestion, such as endoglucanases and β glucosidases [13-18,26-30]. In this respect, a recent study considered host digestive tissues of the lower termite *Hodotermopsis sjostedti *[31]. This research employed five cDNA libraries from various host termite tissues (salivary gland, foregut, midgut, hindgut, and body). A total of 3,548 ESTs were obtained that aligned into 2,366 contiguous sequences. Many genes relating to cellulose and hemicellulose digestion were identified (especially in symbiont-free salivary gland tissue). While these various host and symbiont sequencing efforts to date are both important and provide fascinating information, to our knowledge, no large-scale sequencing studies to date have considered both host and symbiont concurrently from the same termite species.

The termite 'digestome' is defined as the pool of host and symbiont genes that collaborate to achieve high efficiency lignocellulose digestion in the termite gut [5]. Industrial lignocellulose 'pretreatment', alternatively, is a critical step in bioethanol production; it is an early stage, and currently very costly industrial process whereby sugars contained in cellulose and hemicellulose are separated from lignin by non-enzymatic processes [1,32,33]. In this study, our objectives were to: (1) develop a deeper understanding of the digestome of the lower termite *R. flavipes*, and (2) functionally investigate genes with potential relevance to industrial lignocellulose pretreatment. For this purpose, we took a dual host plus symbiont metatranscriptome approach that included large-scale transcriptome sequencing from two separate cDNA libraries; one representing host gut cells and the other hindgut microbial symbionts. Subsequent functional studies of candidate pretreatment enzymes involved efforts to correlate phenoloxidase (laccase and catalase) enzyme activities and gene expression in the termite gut. Our findings reveal: (i) previously unidentified patterns of host and symbiont production of cellulases, hemicellulases, α carbohydrolases, chitinases, and potentially lignases; and (ii) novel host-derived laccase gene products that have potential relevance to industrial lignocellulose pretreatment. The host-symbiont EST database described here (and preliminarily by [5]) represents a significant new genomic resource that will enable robust functional analyses of coevolved host and symbiont digestive arsenals from the same termite species.

## Results

### EST sequencing overview

**Figure 1:**
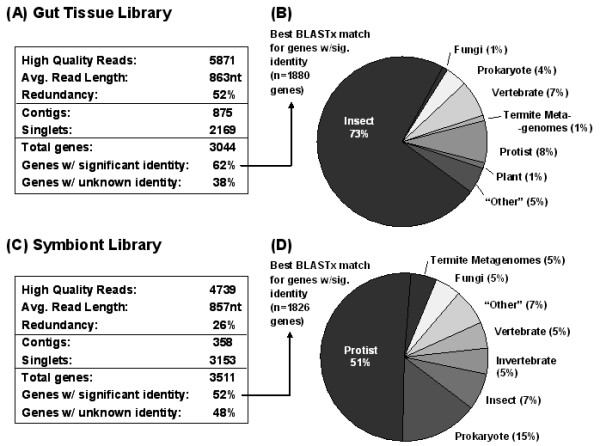
**Expressed sequence tag (EST) sequencing results**. **(a) **and **(c) **present sequencing summaries for the host (termite gut tissue) and symbiont cDNA libraries, respectively. **(b) **and **(d) **are the taxonomic distributions of top BLASTX hits for putative transcripts from the termite gut and symbiont libraries (respectively). Hits producing an E-value < 10^-5 ^were considered significant. The charts illustrate that the majority of transcripts sequenced from the termite gut library were homologous to insect sequences, whereas the majority of transcripts obtained from the symbiont library were homologous to genes previously sequenced from unicellular eukaryotes.

EST sequencing results from host and symbiont cDNA libraries are summarized in Figure 1a,c. A total of 10,610 high-quality ESTs were generated from the 2 libraries. The 5,871 ESTs produced from the host library assembled into 875 contigs and 2,169 singlets to produce 3,044 putatively unique transcripts. In the symbiont EST database, a contig assembly performed using the same parameters identified 358 contigs and 3,153 singlets (3,511 putative transcripts). Thus, the total number of genes identified from the 2 libraries is 6,555. Similarity searches showed that a high proportion of transcripts do not produce any significant match in the NCBI nr database (38% and 48% of host and symbiont libraries, respectively), highlighting the potential of both cDNA libraries for novel gene discovery (Figure 1a,c). The symbiont library contains a higher fraction of uncharacterized sequences (48%) than the termite gut library (38%), reflecting the uniqueness of the symbiont transcript pool and, likely, their unique physiology and metabolism. The annotated transcripts that exhibited significant similarities to known proteins were similar from each library, both in terms of numbers and average sizes of putative genes (Figure 1a,c). Figure 1b,d depict the classification of annotated transcripts based on the organism associated with the top BLASTX hit. As expected, although a minority of microbial sequences is present, >85% of the sequences generated from the termite gut tissue are homologous to known genes previously sequenced from insects or from other invertebrates (Figure 1b). Likewise, while the symbiont library contains a fraction of transcripts matching insect and invertebrate sequences, it contains >85% microbe-homologous sequences (Figure 1d).

### Phylogenetic analyses to investigate taxonomic origins

**Figure 2:**
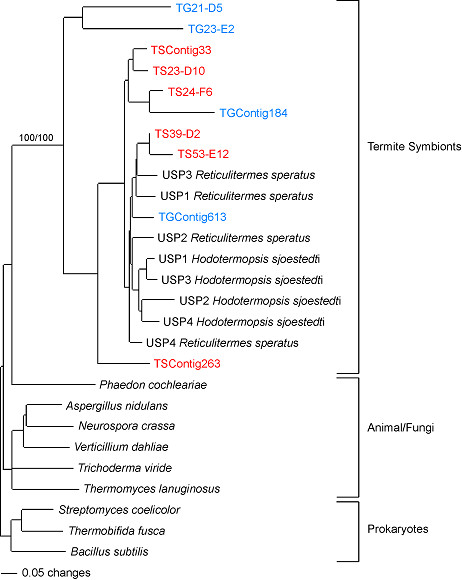
**Protein neighbor-joining phylogeny of glycoside hydrolase family 11 (GHF11)**. The tree was rooted with bacterial GHF11 enzyme sequences and showed that all GHF11 transcripts generated during this study clustered with GHF11 genes previously sequenced from termite symbionts (uncultured symbiotic protist (USP)). The transcripts identified in the termite gut (TG) library are shown in blue, whereas the transcript sequences that originated from the termite symbiont (TS) library are shown in red. Numbers (100/100) above the node represent bootstrap support (1000 replicates). For clarity purposes only relevant bootstrap values are indicated.

Because of apparent library cross contamination, we considered both phylogenetic signal and the library of origin when making taxonomic classifications. In agreement with BLAST analyses, phylogenetic analyses supported that, independent of their cDNA library of origin, a number of GHF family transcripts (for example, GHF1, GHF9 and GHF16) are endogenous to the *R. flavipes *genome (not shown). Likewise, symbiont sequences as suggested by BLAST searching (for example, GHF7, GHF5 and GHF45) all cluster with genes previously attributed to termite symbionts (not shown). As a representative example, Figure 2 depicts the phylogenies of another apparent symbiont-specific family, GHF11. In agreement with previous gut metagenomics research [21], the present analysis shows that irrespective of library all GHF11 sequences generated throughout our study cluster with termite symbiont GHF11 proteins with strong bootstrap support (Figure 2). Additionally, no GHF11 transcripts from either library clustered with fungal or animal sequences, further implying that all GHF11 sequences identified here originate from symbiotic genomes.

### Identification of genes with carbohydrate-active functions

**Figure 3:**
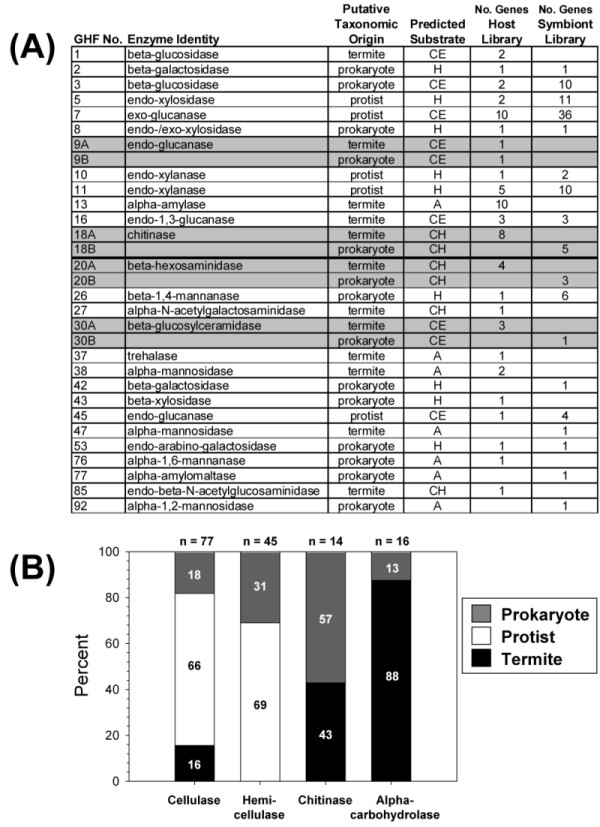
**Distributions of expressed sequence tags (ESTs) encoding glycosyl hydrolase family (GHF) proteins generated from the host gut tissue and hindgut symbiont libraries**. **(a) **A summary of GHF family members with enzyme identities and putative taxonomic origins that were determined based on library of origin and database homology. Shaded rows depict GHF families with representatives from both termite and putative prokaryotic symbionts. CE = cellulase, H = hemicellulase, CH = chitinase, A = α carbohydrolase. **(b) **Bar graph summarizing the taxonomic distributions of cellulase, hemicellulase, chitinase and α carbohydrolase genes among prokaryotic, protist and host genomes. See Additional file 1 for gene by gene summaries and Additional file 3 for Genbank accession numbers.

**Figure 4:**
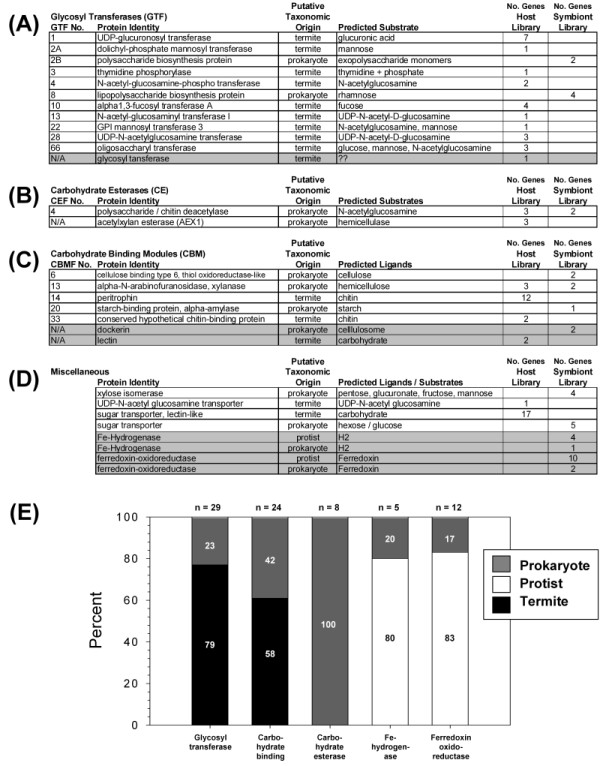
**Distributions of expressed sequence tags (ESTs) encoding (a) glycosyl transferase family (GTF), (b) carbohydrate esterase (CE), (c) carbohydrate binding modules (CBM), and (d) miscellaneous carbohydrate-active and hydrogen-active proteins generated from the host gut tissue and hindgut symbiont libraries**. **(a-d) **Summaries of GHF family members with protein identities and putative taxonomic origins that were determined based on library of origin and database homology. Shaded rows depict gene families not included in the carbohydrate active enzyme (CAZy) database. **(e) **Bar graph summarizing the distributions of glycosyl transferase, carbohydrate binding, carbohydrate esterase, Fe-hydrogenase, and ferredoxin oxidoreductase genes among prokaryotic, protist and host genomes. See Additional files 2 and 5 for gene by gene summaries and Additional files 4 and 10 for Genbank accession numbers.

The termite and symbiont library EST databases were screened for carbohydrate-active enzyme/protein coding sequences. The results of these searches are summarized in Figures 3 and 4, and are presented in more detail in Additional files 1 and 2. All accession numbers for carbohydrate-active enzyme sequences are provided in Additional files 3 and 4. Five categories of carbohydrate-active enzymes/proteins are summarized below.

### Glycosyl hydrolase enzymes

Figure 3a presents GHFs. In all, 27 total families were represented in the host and symbiont sequence pools. The 27 families segregate into the 4 major functional categories of cellulases (GHFs 1,3,7,9,16,30 and 45), hemicellulases (GHFs 2,5,8,10,11,26,42,43 and 53), chitinases (GHFs 18,20,27 and 85), and α carbohydrolases (GHFs 37,48,47,76,77 and 92). Figure 3b summarizes putative taxonomic origins of each family by functional group. This analysis suggests that cellulose digestion is enabled by a three-way collaboration of host plus protist plus prokaryotic symbionts, hemicellulose digestion is accomplished completely by symbionts (protist plus prokaryotic), and both chitin and α carbohydrate digestion are achieved by host plus prokaryotic symbionts.

### Glycosyl transferase enzymes

A summary of identified glycosyl transferase family (GTF) coding sequences is provided in Figure 4a. In all, 10 total subfamilies were represented in the host and symbiont libraries, with 79% and 21% of total unigene sequences being putatively of termite and prokaryotic origins, respectively (Figure 4e). The represented prokaryotic GTFs (families 2 and 8) both encode predicted functions in cell wall biosynthesis. Alternatively, based on significant homology to insect and animal GTFs, the majority of termite-derived GTFs have predicted functions in *N*-acetyl glucosamine modification and/or chitin biosynthesis.

### Carbohydrate esterases

A summary of carbohydrate esterase family members is provided in Figure 4b. These esterases are involved in carbohydrate side chain modification and are considered distinct from the carboxylesterases described later. Predicted functions of the carbohydrate esterases are as chitin deacetylases and acetylxylan esterases. Despite being identified from both the host and symbiont libraries, all of the eight carbohydrate esters identified are apparently encoded by prokaryotic symbionts (Figure 4e).

### Carbohydrate binding modules

A summary of genes encoding carbohydrate binding module family (CBMF) members, as well as dockerins and lectins is provided in Figure 4c. In all, five formal CBM families were represented in the EST dataset (families 6, 13, 14, 20 and 33), of which two are apparently termite derived (14, 33) and all others are apparently from prokaryotic symbionts. Of the termite CBMF members, all are involved in chitin binding; 1 group encodes 12 different peritrophins that are putatively part of the peritrophic membrane secreted around food in the termite midgut. The remaining prokaryotic CBMF members encode putative functions in cellulose, hemicellulose and starch binding. Also included in this category are non-CBMF members; specifically, prokaryotic dockerins that bind cellulolytic enzymes in the secreted cellulosome (two total; Additional file 5) and termite-derived carbohydrate binding lectins that may have immune functions (two total; Additional file 1). Of the 23 total CBMF members identified, 58% are apparently derived from termites and 42% from prokaryotic symbionts (Figure 4e).

### Miscellaneous carbohydrate active moieties

Four additional miscellaneous carbohydrate-active protein families were also identified (Figure 4d). These include prokaryotic xylose isomerases active towards a diversity of monosaccharides (four members), prokaryotic sugar transporters (five members), and termite-produced sugar transporters (two members). Of the termite-derived transporters, there is 1 *N*-acetyl glucosamine transporter potentially involved in peritrophic membrane or gut cuticle biosynthesis, and 17 lectin-like sugar transporters of unknown significance.

### Identification of genes encoding putative lignin degradation, detoxification, and antioxidant functions

**Figure 5:**
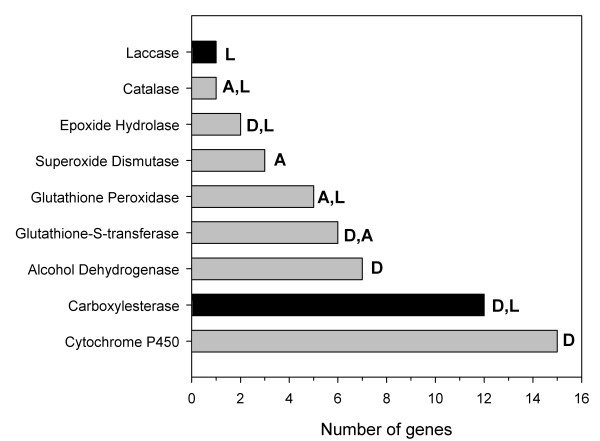
**Summary of potential lignase (L), detoxification (D), and antioxidant (A) coding genes identified from the termite gut library**. No homologous or functionally similar genes were identified from the symbiont library. Black bars indicate gene families investigated in later functional studies (see Figures 6-8). See Additional file 6 for gene by gene summaries and Additional file 7 for Genbank accession numbers.

To examine potential members of lignin degradative pathways, we searched both host and symbiont cDNA library datasets for laccases and peroxidases, which are two gene families known to play roles in fungal lignin degradation (Figure 5 and Additional file 6). In addition, because lignin degradation produces toxic metabolites, both host and symbiont sequence pools were also searched for xenobiotic and antioxidant detoxification enzymes that included alcohol dehydrogenases, catalases, superoxide dismutases, cytochrome P450s, epoxide hydrolases, glutathione *S *transferases, glutathione peroxidases and carboxylesterases. All accession numbers for the putative lignase, detox and antioxidant sequences are provided in Additional file 7. Whereas only 1 putative peroxidase gene was identified in the symbiont EST pool (accession numbers FL643288, FL645697), the termite gut library sequence pool was found to contain 52 enzyme coding genes putatively associated with lignin degradation or xenobiotic metabolism/detoxification (Figure 5 and Additional file 6). With regard to laccases, the presence of laccase transcripts in the termite library, but not the symbiont library, supports that termite gut cells produce laccases (six clones representing one putative gene; accession numbers FL639514, FL640712, FL635040, FL635071, FL635132, FL635524). The termite laccase contig also has highest homology to other insect laccases, supporting that its origin is from the host genome (Additional file 8).

Carboxylesterases are another potentially important group of termite-derived enzymes identified through the current work. A total of 12 distinct carboxylesterase genes were identified from the termite gut gene pool (Figure 5), all of which were absent from the symbiont library and had highest homology to insect carboxylesterases. A search for signal peptides at the N-termini of predicted protein sequences suggests that termite gut cells secrete laccases, catalases, cytochrome P450s, carboxylesterases, superoxide dismutases, epoxide hydrolases, and glutathione peroxidases (Additional file 6); however, because of the known membrane-bound nature of P450 proteins, they are not likely to be secreted.

### Gut phenoloxidase activity, phenoloxidase induction by lignin feeding, and laccase gene expression

**Figure 6:**
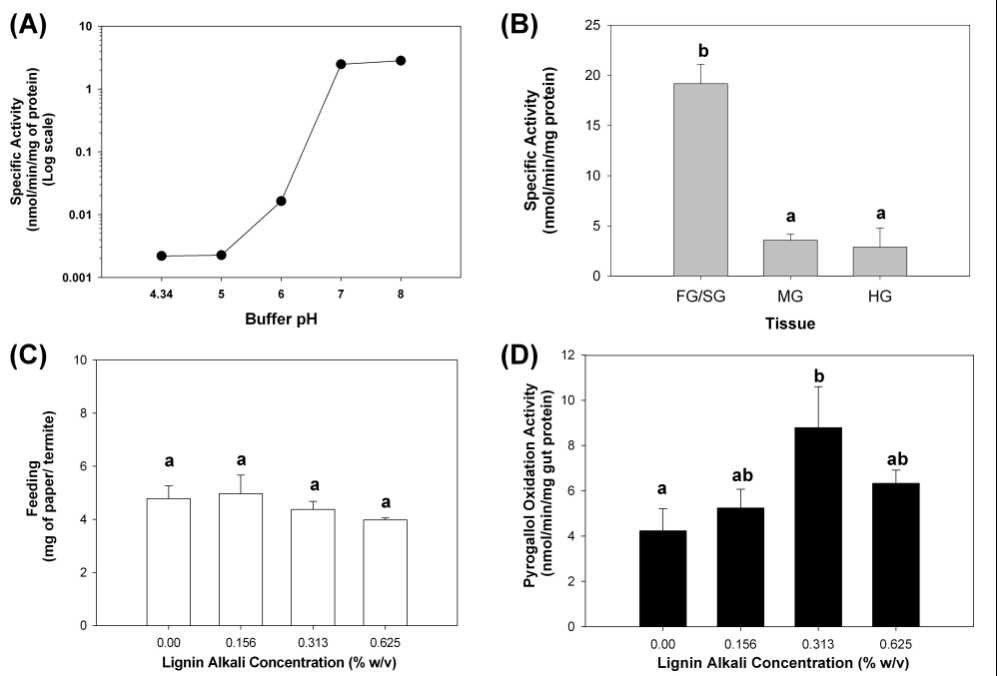
**Functional analysis of phenoloxidase activity towards the model substrate pyrogallol**. **(a) **pH dependence of pyrogallol oxidation determined using whole guts of worker termites. **(b) **Distribution of pyrogallol oxidation activity across the termite gut using 14,000 *g *supernatants from homogenized tissues of the foregut plus salivary gland (FG), midgut (MG), and hindgut (HG). **(c) **Feeding by live termites for 7 days on papers treated with lignin alkali at concentrations ranging from 0% to 0.625% w/v. **(d) **Pyrogallol oxidation activity in whole termite guts after feeding on various concentrations as depicted in **(c)**. Bars with the same letters are not significantly different by least significant difference (LSD) t tests (*P *< 0.05).

The identification of a novel laccase (EC 1.10.3.2) gene from the host library prompted us to investigate phenoloxidase activity towards the model laccase substrate pyrogallol in a colorimetric microplate assay (Figure 6). All results shown are the average ± standard error determined from three colonies. pH-dependent pyrogallol oxidation activity was highest at pH 7-8 (Figure 6a), which partially encompasses the physiological pH range of 6-7 measured previously in the *R. flavipes *gut [34]. Next, the tissue distribution of pyrogallol oxidation activity was determined at the optimal pH of 7 (Figure 6b). In strong agreement with laccase gene expression (see below), pyrogallol oxidation activity was approximately sixfold higher in the foregut plus salivary gland than in the midgut and hindgut. This activity profile did not correlate with catalase, carboxylesterase, or reference gene expression (see below). Finally, pyrogallol activity was investigated after feeding live termites for 7 days on cellulose filter papers treated with partially depolymerized lignin (called 'lignin alkali'). Although no significant differences in feeding occurred between controls and the different lignin bioassay concentrations (Figure 6c), there was a significant induction of gut pyrogallol oxidation activity associated with one lignin bioassay concentration (0.313%; Figure 6d).

**Figure 7:**
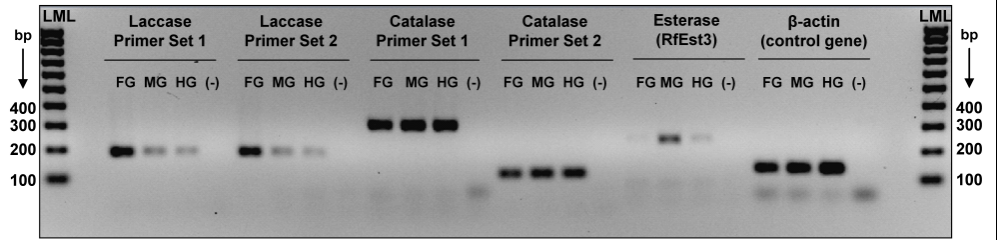
**Laccase, catalase, RfEst3 esterase, and β actin (as a control) gene expression in *Reticulitermes flavipes *worker guts as determined from 35 cycles of conventional polymerase chain reaction (PCR) amplification**. Shown is a negative image of a representative agarose gel with equal loadings of PCR products for the four genes. Lane labels are as follows: FG, foregut plus salivary gland; MG, midgut; HG, hindgut; and (-), no template control. Two primer sets were tested for the laccase and catalase genes, and one primer set for esterase and β actin. A 100 base pair (bp) low molecular weight ladder (LML) is shown at the left and right. See Additional file 9 for PCR primer sequences.

Laccase (contig 659) and catalase (contig 230) gene expression across the *R. flavipes *gut, relative to the *RfEst3 *carboxylesterase (contig 275) and β actin as a control gene, is shown in Figure 7. Two laccase and two catalase primer sets were tested; both primer sets for each gene yielded identical results. Laccase expression was highest in the foregut plus salivary gland, lower in the midgut, and lowest in the hindgut. Catalase and β actin expression were uniform across gut regions; in contrast, RfEst3 expression was highest in the midgut.

## Discussion

### Overview

The broad goals of this research were to obtain robust pools of host and symbiont lignocellulase and other carbohydrate active genes from the *R. flavipes *gut, and to begin functional investigations of candidate genes with potential roles in industrial lignocellulose pretreatment. Two cDNA libraries were created for these purposes: (i) a normalized host cDNA library prepared from gut tissues cleared of symbionts and (ii) a non-normalized library prepared from hindgut symbionts. The rationale for taking these approaches was that the normalization process would maximize representation of host genes in the host library (from our results it is clear that symbionts were not entirely removed from guts upon dissection), while genes represented in the non-normalized symbiont library would correspond to the relative abundance (and presumably importance) of the hindgut symbiota. Preliminary sequencing summaries from this work are presented in Scharf and Tartar [5]. The currently reported sequence results presented in Figures 3, 4, 5 are the results of new database identity searches in April 2009 after several new large-scale sequencing projects were deposited in Genbank [23,24,31].

In addition to identifying a robust complement of host and symbiotic protist genes, numerous genes with strong prokaryotic homology were also identified. Because prokaryotic genes are known to be polyadenylated [35], our cDNA synthesis strategy involving isolation of polyA RNA with 'oligo-dT' priming resulted in the inclusion of apparent prokaryotic cDNAs in our libraries. Thus, although a large fraction of prokaryotic transcripts were likely not captured, the prokaryotic genes identified here are likely to be legitimate polyadenylated prokaryotic genes. However, it is also possible that prokaryotic symbiont genes have assimilated into host and/or protist genomes [36-38], and/or that some microbial symbiont sequences already deposited in Genbank may have been misannotated. For example, with respect to the latter point, previous metatranscriptome sequencing projects on termite hindgut environmental cDNA did not consider the possibility that prokaryotic transcripts can be polyadenylated and enriched via polyA RNA purification protocols. Additional efforts beyond the scope of the current work will be required to resolve these potential assimilation and annotation issues.

With respect to functional analyses, we focused on previously unknown carboxylesterase and phenoloxidase coding genes ([39] and this work) with potential relevance to industrial lignocellulose pretreatment [33]. Both of these enzyme families are considered more relevant for pretreatment than cellulases or hemicellulases, which are more relevant in downstream carbohydrate depolymerization processes that immediately precede simple sugar fermentation [33]. Additionally, cellulase and hemicellulase genes/activities in *R. flavipes *are already well documented ([17,18,30,40-43] and the present work). Fungal carboxylesterases known as 'feruloyl esterases' solubilize hemicellulose by cleaving ester bonds between hemicellulose and monolignols and/or phenolic acids [44-47], and thus the host carboxylesterases identified here represent potentially important pretreatment enzymes. Based on the well established importance of fungal laccases and related enzymes in lignin degradation [48,49], phenoloxidases/laccases are also relevant to industrial lignocellulose pretreatment.

Finally, the host and symbiont sequence pools resulting from this work constitute an important new genomic resource. This resource has potential applications in microarray, heterologous/recombinant protein expression, and biochemical characterization studies that can provide more definitive insights into digestome function and host-symbiont collaborative digestion than the current sequencing work. In the sections that follow, details, inferences and applied impacts of the current findings in relation to host-symbiont collaborative digestion and biofuel production are discussed.

### Comparisons of carbohydrate-active genes from host and symbiont

#### Overview

Despite apparent symbiont contamination in the host library, >85% of carbohydrate active sequences from termite gut library had significant homology to translated genes of animal origin. Likewise, despite apparent host contamination, more than 85% of the sequences from the symbiont library with carbohydrate-active database matches were found to be most similar to predicted proteins from microbes. However, interestingly, only 5% of these transcripts were directly related to sequences from known termite symbionts or metagenome/transcriptome databases, highlighting the poor representation of close homologues within the extant public protein database. It is possible that with deeper sequencing, greater coverage, and specific prokaryote targeting, more homologues to existing metagenome sequences could be identified.

#### Contaminating sequences

Specific examples of contaminating sequences that we observed are as follows. In the gut library such transcripts included α and β tubulin homologues (data not shown) similar to sequences recently produced from the parabasalian termite symbiont *Spirotrichonympha leidyi *[50], as well as GHF7 glycosyl hydrolases (Figure 3; Additional file 1) previously shown to be associated with eukaryotic gut symbionts [20,29]. Other potentially contaminating sequences identified from the gut library were from unicellular organisms such as fungi, protists and prokaryotes, as well as sequences homologous to others previously identified from termite metagenome projects [20,21]. However, by noting phylogenetic BLAST homologies of specific genes and gene families from each library, we have been able to deduce the likely origins of certain gene families.

Although hypotheses about lateral gene transfer and potential misannotation of archived database sequences cannot be dismissed, our conclusion from the present sequence composition findings is that host and symbiont are intimately connected and therefore cannot be completely excluded from each other and sampled/studied independently. For example, a subset of prokaryotic symbionts in *Reticulitermes *are attached to the gut wall [51,52]. This phenomenon is concordant with our observation that the termite gut library contained a fraction of sequences of prokaryotic origin. Also, protist gut symbionts are now known to contain co-evolved bacterial endosymbionts [23,24,53-55], which, because of possible assimilation, may limit the ability to distinguish host vs. protist and bacterial endosymbiont genes.

#### Phylogenetic analyses

The tree presented in Figure 2 is representative of all GHF phylogenetic analyses that were conducted. These analyses are extremely concordant with the glycosyl hydrolase family (GHF) gene listing available in the CAZy database . The CAZy lists show that GHF1, GHF9 and GHF16 genes have been widely sequenced from insects (including termites) but have never been reported in eukaryotic termite symbionts. In contrast, GHF7, GHF11 and GHF45 have been sequenced in fungi and termite symbionts but they are virtually absent from metazoan genomes [21]. In addition to GHF enzymes (Figure 3), significant differences in gene content of many other carbohydrate-active and hydrogen metabolism enzymes were also observed when comparing the two libraries (Figures 3 and 4). Despite these trends, we took the more conservative approach that considered both phylogenetic signal and the library of origin when making taxonomic classifications. If correct, our findings provide seminal evidence of a tripartite collaboration of host plus protist plus prokaryote in cellulose digestion and exclusive dependence on symbiotic fauna (prokaryote plus protist) for hemicellulose digestion.

#### Symbiont GHFs

Based on the hypothesis that the frequencies of ESTs in non-normalized cDNA libraries are positively correlated with gene expression levels, consistent with Todaka *et al*. [20], the current data indicate that *R. flavipes *gut symbionts overwhelmingly produce GHF7 enzymes. This GHF family is mostly known for its cellobiohydrolase (exoglucanase) and endoglucanase activities against microcrystalline cellulose [16,20,56]. The symbiont library sequence pool also suggests that gut symbionts abundantly produce GHF3, GHF5, GHF10, GHF11, GHF26 and GHF45 hydrolases, whereas all other catalytic domains were observed more sparingly (Figures 3 and 4). Although some of these apparent symbiont sequences were also identified in the host library, and assuming that horizontal gene transfer and previous database misannotation have not occurred, homology and phylogenetic analysis results confirm that these GHF sequences are all symbiont derived. These symbiont library findings are concordant with another metatranscriptome sequencing effort performed on the *R. speratus *symbiotic protist community, which revealed that members of GHF7 were the most extensively expressed enzymes [20]. These similarities suggest that both *R. flavipes *and *R. speratus *symbiont populations possess a similar glycohydrolase arsenal, composed predominantly of GHF7 hydrolases and a number of additionally important cellulase and hemicellulase families (including GHF3, GHF5, GHF11, GHF26 and GHF45). However, the current study revealed at least thirteen GHF families from both *R. flavipes *libraries that were not identified in *R. speratus*, specifically GHF2, 9, 13, 16, 18, 20, 27, 30, 37, 38, 42, 47 and 53. Additionally, no laccases or other potential lignase enzymes were identified in *R. speratus *hindgut symbiont library as they were here from the *R. flavipes *host library (see below). Another potentially important set of symbiont genes identified in the current work encode prokaryotic dockerin proteins. Dockerins are important components of prokaryotic cellulosomes, which are secreted complexes of enzymes and proteins that synergistically collaborate in plant cell wall depolymerization [57]. No dockerin genes have been reported previously from lower termites.

#### Carbohydrate active host genes

The gene expression profile of the host termite gut cells is not characterized by an overly abundant domain (Figures 3 and 4, Additional file 1); however, this may be related to the fact that the host library was normalized. Important carbohydrate-active transcripts from the termite host are mainly associated with cellulose degradation (GHF1, 9, 16); chitin metabolism, which is an important antifungal and cuticle melanization pathway in insects (GHF18, 20, 27, 85); depolymerization of α-linked carbohydrate polymers (GHF13, 37, 38, 47); glycosyl transferases which are likely to play roles in gut chitin/cuticle biosynthesis (GT1, 2, 3, 4, 10, 13, 22, 28, 66); carbohydrate binding proteins/modules also involved in cuticle/peritrophic membrane biosynthesis (CBM 14,33), a carbohydrate binding lectin (2 genes); and finally, lectin-like sugar transporters (17 genes). No such genes were reported from the *R. speratus *hindgut symbiont library [20]. In agreement with previous findings [13,16,17,19-21,27,31], the host library dataset strongly suggests that termite gut cells produce unique glycoside hydrolases that complement activities derived from symbionts. Examples of such gene families include GHF1, GHF9 and GHF16, which are predicted to encode β glucosidase (GHF1) and endoglucanase (GHF9, 16) activities, and which accounted for 6.5%, 7.5% and 10.5% of all GHF-domain-containing clones, respectively, in the host library. Of the termite host-specific glycohydrolases, GHF9 endoglucanases and GHF1 β glucosidases could play strong collaborative roles to liberate glucose from cellulose, as they do in *Trichoderma *engineered for maximal cellulase depolymerization in bioethanol production from second-generation feedstocks [33,58].

### Candidate lignin degradation, detoxification and antioxidant factors

#### Overview

Lignin degradation is a critical first step in lignocellulose digestion as it allows for the dissociation of lignin, cellulose and hemicellulose, making carbohydrate polymers available for enzymatic digestion [3,5,33]. This step remains poorly understood in termites and other wood-feeding insects, but it has been well characterized in lignin-degrading fungi, where oxidative enzymes, including laccases, manganese peroxidases and lignin peroxidases have been shown to play major roles in delignification [3,48,49]. A recent study demonstrated indisputably that lignin is degraded on a scale of hours within the guts of wood-feeding insects, including the lower termite *Zootermopsis angusticollis *[59]. Many previous studies over several decades have also provided evidence of lignin degradation capabilities in termite guts, including *R. flavipes *[12,34,60-67]. No candidate lignase genes from termites have been identified prior to the current work.

Several genes identified in the present study may play direct roles in either lignin degradation or protection from reactive oxygen species and other toxic metabolites generated during lignin degradation. BLAST analyses demonstrated that all genes listed in this category have homologues in insects, and they were identified exclusively in the host library, supporting that they are endogenous to the host genome. The search for signal peptides at the N-termini of predicted protein sequences and subsequent 5' RACE efforts [Coy NR, Salem TZ, Denton JS, Kovaleva E, Liu Z, Campbell J, Davis D, Buchman G, Boucias DG, Scharf ME, unpublished results] suggest that termite gut cells secrete laccases, catalases, esterases, cytochrome P450s, superoxide dismutases, epoxide hydrolases, and glutathione peroxidases (Additional file 6). However, as reviewed previously [68] cytochrome P450 proteins are usually retained intracellularly in insect cells where they remain anchored in the endoplasmic reticulum or mitochondria. Despite their expected subcellular distributions, insect P450s are adept at catalyzing *O*-demethylation reactions [68] such as the lignin side chain oxidations reported by Geib *et al*. [59]. All other enzymes (laccases, catalases, carboxylesterases, superoxide dismutases, epoxide hydrolases and glutathione peroxidases) are likely to be secreted in the gut lumen [39] and therefore are promising candidates to test for activity against lignin, its phenolic acid monomers, and/or its metabolites.

#### Laccases and catalases

After obtaining sequence data indicating the presence of laccase gene expression in the *R. flavipes *gut, we undertook functional studies using the model substrate pyrogallol. Pyrogallol is a proven substrate for fungal laccases that are known to degrade lignin [49]. In addition to observing an induction of pyrogallol activity from live termites after lignin feeding (Figure 7), laccase activity has also now been confirmed towards several other laccase model substrates, including hydroquinone and dimethoxyphenol, using both salivary gland homogenates and recombinant laccase proteins [Coy NR, Salem TZ, Denton JS, Kovaleva E, Liu Z, Campbell J, Davis D, Buchman G, Boucias DG, Scharf ME, unpublished results]. Correlated laccase gene expression and phenoloxidase activity in the foregut/salivary gland region (Figures 6 and 7) and the presence of a signal peptide in the laccase translation product [Coy NR, Salem TZ, Denton JS, Kovaleva E, Liu Z, Campbell J, Davis D, Buchman G, Boucias DG, Scharf ME, unpublished results] are consistent with the hypothesis that laccase protein is synthesized in the salivary gland and secreted into the foregut. This expression pattern is in agreement with the relatively high oxygen content occurring in the foregut region [61], and is similar to that of host-secreted salivary *Cell-1 *endoglucanase of *R. flavipes *[17,30]. Catalases, while known to possess phenoloxidase activity [69,70], were not considered further due to the lack of correlation observed between catalase gene expression and phenoloxidase activity (Figures 6 and 7).

Laccases have long been associated with phenoloxidase reactions required for insect cuticle tanning (for example, [71,72]); however, strong laccase gene expression and activity in the midguts and salivary glands of herbivorous insects [73,74] are suggestive of digestive roles. The recombinant *R. flavipes *laccases noted above is essentially inactive against tyrosine, dopamine and other melanin precursors, which is consistent with the hypothesis that it is not an insect cuticle laccase [Coy NR, Salem TZ, Denton JS, Kovaleva E, Liu Z, Campbell J, Davis D, Buchman G, Boucias DG, Scharf ME, unpublished results]. Additionally, the *R. flavipes *laccase sequence has significant homology to ESTs sequenced from a salivary gland cDNA library of the termite *Hodotermopsis sjostedti *(Genbank accession numbers DC232380, DC235488), and also, host-derived laccase gene expression has now been putatively identified from the gut of a related lower termite, *C. formosanus *[75].

Insect laccases are phylogenetically close to fungal laccases [76], which are known for being prominently responsible for lignin degradation in white rot fungi [3,6,49] and possibly termite-cultivated fungi [77]. Sequence alignments of the translated laccase contig assembled from the present sequence data with insect and fungal laccases indicate that the termite laccase is very similar to both insect and fungal laccases (Additional file 8). The emerging evidence of endogenous laccase gene expression in termite guts is both interesting and worthy of further pursuit given that (i) lignin degradation occurs in termite guts [59], and (ii) no laccase genes have been identified in any symbiont sequencing projects ([20-24,59] and the present work).

#### Carboxylesterases

Carboxylesterases are another potentially important group of termite-derived digestive enzymes that were identified through the current work. Although previous studies have investigated termite esterase activity [78-83], to our knowledge, this report and [39] provide seminal evidence of esterase gene expression in termite guts. All carboxylesterase genes were sequenced from only the host library, they are expressed in symbiont-free tissues [39], and they have significant homology to insect carboxylesterases [39]; therefore they are believed at this time to be encoded in the termite genome and produced by termite gut cells. In agreement with our identification of 12 host-derived carboxylesterase genes in the present study, functional analysis of gut esterase activity revealed strong levels of activity and diverse isoforms by native polyacrylamide gel electrophoresis (PAGE) analysis with an esterase model substrate [39]. Full-length gut esterase gene sequences and more detailed functional biochemistry results are presented in [39].

Many insect esterases have well defined biological functions, such as xenobiotic, lipid, acetylcholine, and juvenile hormone metabolism [84]. However, while being extremely efficient at metabolizing model substrates such as naphthyl and *p*-nitrophenyl esters, the vast majority of insect esterases have largely undefined functions. This latter category of esterases is referred to as the 'general esterases'. Our hypothesis [5,39] is that termite gut carboxylesterases are potentially important in cleavage of lignin and lignin monomers from hemicellulose [2,3,5], as well as in the lignin side chain oxidations identified recently in the termite gut [59].

#### The *R. flavipes *digestome sequence pool as a new genomic resource

The host-symbiont sequence dataset reported here represents an excellent genomic resource for addressing a number of important applied research topics; most importantly, collaborative host-symbiont digestion and development of novel biocatalysts for use in sustainable bioethanol production. As a first step toward addressing both topics, microarrays can now be developed that contain the entire complement of host and symbiont genes. Using digestome microarrays, termite colonies can be fed different lignocellulosic materials or second-generation bioethanol feedstocks [85] and then assessed for global differences in host plus symbiont gene expression. It is anticipated that this approach will reveal vastly different complements of host and symbiont gene expression in response to different lignocellulose diets, as well as which complements of enzymes are most relevant to target for use in digestion/depolymerization of specific feedstocks. Another approach to resolve sequence origin will be to probe microarrays that contain host and symbiont genes together with enriched symbiont RNA fractions isolated from the hindgut lumen.

Once relevant digestive genes are identified, enzyme blends can be functionally expressed and tested as biocatalysts for collaborative digestion of natural materials and agricultural/forestry feedstocks. Approaches to produce recombinant enzymes can include heterologous expression in prokaryotic or eukaryotic systems. Examples of prior successful efforts to functionally express termite and symbiont digestive enzymes include GHF9 endoglucanases from *C. formosanus *and *Coptotermes acinaciformis *in *Escherichia coli *[75,86,87]; GHF5, GHF9 and GHF45 cellulases from *Nasutitermes *hindgut bacteria in *E. coli *[21], and *R. speratus *protist symbiont GHF7 exoglucanases in *Aspergillus oryzae *[88]. For industrial biorefinery applications, we hypothesize that coevolved host and symbiont enzymes from the same system, that is, the *R. flavipes *digestome, will be more efficient than a mix of enzymes from different systems such as termite symbiont and *Trichoderma*. In this respect, our group has recently functionally expressed highly active forms of a host GHF9 endoglucanase and symbiont GHF7 exoglucanase identified in the current study (Figures 2 and 3) and previously [17,18], as well as a host laccase and GHF1 β glucosidase identified in the present study. For expression of these proteins, we employed a baculovirus insect expression system that enables large-scale production of recombinant proteins with the added benefit of eukaryotic transcriptional and pre/post-translational processing [89].

Finally, the inability to completely physically separate host from symbiont suggests that future efforts to investigate host-symbiont lignocellulose digestive gene expression would be better approached by the parallel sequencing of host and symbiont transcriptomes in a comprehensive meta-analysis. In this respect, the host and symbiont databases described here and previously [20-25,31] will provide a powerful bioinformatic scaffold to assist future efforts in this area, as well as proteomic efforts.

## Conclusion

The research presented here took a unique host plus symbiont EST sequencing approach to identify host and symbiont contributions in collaborative lignocellulose digestion by termites. EST sequencing from host and symbiont cDNA libraries provided >10,000 ESTs that aligned into 6,555 putative genes with an immense diversity of functions. Each library contained a significant proportion (approximately 15%) of apparent cross contaminating sequences from its opposite fraction, suggesting that library composition alone cannot provide conclusive information on host-symbiont collaboration. Because of the apparent library cross contamination (and assuming that assimilation and database misannotation did not occur), we considered both phylogenetic signal and the library of origin (host or symbiont) when making taxonomic classifications based on sequence identity.

Most importantly, glycosyl hydrolase sequence analyses revealed an apparent three-way collaboration between host and protist plus prokaryotic symbionts in cellulase production, a two-way collaboration between protist plus prokaryotic symbionts for hemicellulase production, and a two-way collaboration between host plus prokaryotic symbionts in chitinase and α carbohydrolase production. Searches for other carbohydrate-active moieties indicated that glycosyl transferase, carbohydrate binding, and sugar transport functions are shared between the termite host plus prokaryotic symbionts, whereas acetyl side chain carbohydrate esterase activities are encoded by prokaryotic symbionts. Other highly relevant prokaryotic genes such as cellulosome dockerins that play known roles in facilitating cellulose digestion were also identified, supporting that these activities are entirely symbiont-derived.

The gut (host) library exclusively revealed a number of phenoloxidase and peroxidase genes that play potential roles in lignin degradation/depolymerization, as well as other detoxification and antioxidant genes that may protect termite cells and symbionts from damage by lignin degradation products. The gut (host) library also exclusively revealed a number of carboxylesterase genes with potential roles as lignin depolymerizing esterases or feruloyl esterases that solubilize hemicellulose by cleaving carboxyl ester bonds between hemicellulose (pentose) sugars and lignin or monolignols [39]. Examination of phenoloxidase activity also revealed, for the first time, significant pyrogallol oxidation activity in the termite gut, induction of pyrogallol activity by lignin feeding, and strong agreement between laccase gene expression and pyrogallol oxidation activity. More detailed results of carboxylesterase, laccase and various cellulase catalytic activities, obtained using recombinant proteins and other functional/genomic approaches will be the focus of forthcoming reports.

It is well established that lignocellulose digestion in the termite gut is a highly efficient process (for example, [65]). Because lignocellulose is composed of 40% cellulose, 25% hemicellulose, and 20% lignin [32], it would be expected that a proportional diversity of digestive factors specific to these three components should exist in the termite gut digestome. In this respect, the dual sequencing approach presented here revealed a large and diverse complement of host and symbiont genes with significant links to lignocellulose digestion. The total number of candidate lignocellulases identified here was 171 (45% cellulase, 26% hemicellulase, and 29% lignase/detox candidates). In addition to identifying thousands of genes with both well defined and undefined GO functions, including many potential digestive gene families not revealed by prior symbiont sequencing projects, the present research has also revealed previously unseen patterns of host and symbiont digestive gene expression. While the present work clearly has limitations, these findings are unprecedented; not only do they offer the first concurrent, whole-digestome glimpse into host-symbiont commensalism/mutualism in termites, they also provide a new genomic resource for developing coevolved host and symbiont biocatalysts for use in biomass-to-bioethanol applications.

## Methods

### Biological samples

All termites used in this study were verified as *R. flavipes *using a combination of 16S rDNA sequence using genomic DNA obtained from termite heads [90] and soldier morphology. Termites were collected on the University of Florida campus (Alachua County, FL, USA) and held in the laboratory on a pine wood and paper diet for 3-6 months before use.

### Construction of the termite gut (host) cDNA library

Approximately 2,000 guts from *Reticulitermes flavipes *worker termites from 5 colonies, with attached salivary glands, were dissected and cleared of symbionts to yield approximately 1000 mg of tissue. Dissected guts were cleared of symbionts by placing individual guts into a droplet of phosphate buffered saline (PBS; pH 7), opening the paunch with dissecting scissors, rinsing in a second droplet of PBS, and then by blotting the gut on a piece of laboratory tissue paper with dissecting forceps. Guts were stored for 1-2 months at -80°C until RNA isolation. Total RNA was isolated using the SV Total RNA kit (Promega; Madison, WI, USA) and mRNA purified using the Oligotex mRNA mini-kit (Qiagen; Valencia, CA, USA). First strand cDNA was synthesized from mRNA using SMART technology [91] as part of the SMART cDNA Library Construction kit (Clontech; Mountainview, CA, USA). Next, the cDNA was normalized using proprietary technology from Clontech that reduces abundant transcripts, enriches for rare transcripts, and also enriches for full-length mRNAs [92]. Normalization included cDNA denaturation/reassociation, treatment by duplex-specific nuclease [93] and amplification of the normalized fraction by polymerase chain reaction (PCR). The normalized cDNA pool was end digested and ligated into the pDNR-Lib vector (Clontech) then transformed into BH10B competent cells (Clontech). To obtain enough high-quality plasmid for library screening and for long-term storage, the library was amplified and cultured to yield a final titer of 4.2 × 10^6 ^cfu/ml.

### Construction of the symbiont fauna cDNA library

The digestive tracts from 300 mature workers were dissected and released hindgut luminal contents were collected into a small drop of phosphate buffered saline. The gut contents, pooled in a microcentrifuge tube on ice, were centrifuged at 13,000 rpm at 4°C for 15 min. Total RNA was extracted from cell pellets using the SV Total RNA Isolation System (Promega). The ability of lysis buffer to disrupt the microbiota in the pellets was confirmed microscopically. Approximately 100 μg fresh (never frozen) total RNA was precipitated with ethyl alcohol/ammonium acetate/glycogen and then processed through the PolyA Purist kit (Ambion; Austin, TX, USA). cDNA was synthesized immediately from the polyA RNA (approximately 1 μg) fraction and ligated into the pDONR 222 plasmid utilizing the CloneMiner cDNA Library construction kit (Invitrogen; Carlsbad, CA, USA). Plasmid preparations were electroporated into competent ElectroMAX DH10B T1 phage-resistant cells (Invitrogen). The library was amplified and cultured to yield a final titer of 1.5 × 10^7 ^cfu/ml.

### Isolation of clones for sequencing

For sequencing, frozen library aliquots were scraped and placed into 1 ml ice cold 1 × PBS. The resulting aliquots (1-10 μl) were diluted further into 1 ml SOC media [94] and plated on LB-agar plates containing 41 μg/ml chloramphenicol (gut library) or LB-agar containing 60 μg/ml kanamycin (symbiont library). Plates were inoculated with variable volumes of the diluted library mixture to achieve optimal spacing. Streaked plates were incubated at 37°C for 16-18 h. Well spaced colonies were selected at random and placed into 500 μl LB media with 8% glycerol containing either 34 μg/ml chloramphenicol (gut library) or 50 μg/ml kanamycin (symbiont library). After inoculation, liquid cultures were shaken (250 rpm) at 37°C for 16-18 h. Cultures were frozen at -80°C until plasmid isolation and sequencing as described below.

### Sequencing and sequence analysis

Both libraries were considered independently throughout the sequencing and analysis processes. Sequencing was performed by the Interdisciplinary Center for Biotechnological Research (ICBR) at the University of Florida, and it consisted of single pass reactions from the 5' end of the cDNA, using the M13 forward primer. Trace files were screened to identify and eliminate vector sequences and low-quality reads, using the ICBR software package 'Finch-Suite' (Geospiza Inc., Seattle, WA, USA). Sequences shorter than 150 base pairs (bp) were also removed from the final sequence pools. The remaining high-quality sequences were used as inputs for contig assemblies performed using CAP3 [95] with default parameters (including overlap cut-off values fixed at 30 bp and 75% identity). The clustered sequences were then annotated based on similarity searches performed by batch BLAST analyses using the Greengene interface . BLAST analyses included searches against the non-redundant (nr) NCBI protein database (BLASTX), as well as against the Conserved Domain Database. An E-value cut-off of 10^-5 ^was used for all BLAST searches, and in >95% of instances, both identities and taxonomic origins were supported by at least the top 10 BLAST hits. Sequences associated with carbohydrate catabolism were classified according to the CAZy nomenclature (; [96]). Identification of signal peptides at the N-terminus of putative enzymes was performed using the SignalP program [97].

### Phylogenetic analyses

Sequences predicted to encode glycoside hydrolases were translated *in silico *and aligned with homologous sequences accessed from the CAZy databases. Alignments were performed using CLUSTALX [98]. Phylogenetic relationships were reconstructed in PAUP* (Sinauer Associates, Sunderland MA, USA) using maximum parsimony and distance (neighbor joining) models, with default parameters. Support for relationships was assessed by bootstrap analyses (1000 replicates).

### Phenoloxidase functional analyses

#### Protein isolations

All manipulations were performed on ice. For tissue localization studies, 25 termite worker guts from 3 separate colonies were removed and dissected into the 3 regions of foregut plus salivary gland, midgut and hindgut. Each gut region preparation was homogenized using a Tenbroeck glass homogenizer in potassium phosphate buffer (0.1 M, pH 7.6), and then centrifuged for 15 min at 14,000 *g *and 4°C. The supernatant was saved for assays and the pellet discarded. Whole gut preparations were prepared identically, except that 25 guts were dissected and homogenized. Protein content of protein preparations was estimated by a microplate Bradford assay (Bio-Rad; Hercules, CA, USA) using bovine serum albumin as a standard and corresponding buffers as blanks.

#### Phenoloxidase assay

Oxidation of the model substrate pyrogallol to the product purpurogallin was investigated using the method of Chance and Maehly [99], modified for a 96-well microplate format. Each assay reaction (250 μl) contained 168 μl nanopure water, 25.6 μl potassium phosphate (100 mM, pH 4-8), 12.8 μl of 0.5% hydrogen peroxide (not necessary for activity; Aldrich, Milwaukee, WI, USA; prepared fresh in water), 25.6 μl of 5% pyrogallol solution (Sigma, St Louis, MO, USA; prepared fresh in water), and 8 μl protein preparation. For assays, protein preparations were first added to microplates on ice. All reagents were then combined as described above into a master mix. Assays were started by adding 242 μl master mix solution to reaction wells using a multichannel pipette. Assays were conducted at room temperature and read kinetically at 420 nm for a total of 30 min. Blank reactions contained an equivalent volume of potassium phosphate in place of protein preparation. Specific activity in nmol/min/mg was determined from the linear portions of reaction curves using an extinction coefficient of 24.7 mM/cm [99] and by correcting for protein content. The equation used to calculate specific activity was as follows: (extinction coefficient) × (velocity per min) × ((protein concentration in mg/ml)/(protein dilution factor in assay)). Feeding assays were also conducted that investigated phenoloxidase activity after termite feeding for 7 days on filter papers treated with three serial concentrations of lignin alkali extract (0.625, 0.313, 0.156% w/v; Sigma). All results are summarized from three protein preparations, each assayed in triplicate. Statistical analyses consisted of analysis of variance (ANOVA) with least significant difference (LSD) t tests for mean separation.

#### PCR to verify gene expression

All PCR primer sequences are provided in Additional file 9. The stable expression of the reference gene β *actin *across gut regions was validated previously [17]. PCR primers were designed with specificity to unique sequence regions of the target genes, and to produce products in the 100-300 bp range. PCR primers were designed using Primer3 [100]. cDNA from the foregut plus salivary gland, midgut and hindgut regions served as the template for PCR. Gut dissections and homogenizations were performed as described above, using RNA lysis buffer (Promega) in place of potassium phosphate. cDNA was synthesized from the total RNA of 25 individual gut regions per experimental replicate. Total RNA and cDNA were obtained using the SV total RNA isolation kit (Promega) and the iScript cDNA Synthesis Kit (Bio-Rad), respectively, following manufacturer protocols. PCR reactions contained equal template loadings and proceeded for 35 cycles. Control reactions were conducted in the absence of cDNA template. PCR products were viewed on 1.5% agarose gels and imaged using a Gel-Doc 2000 imaging system (Bio-Rad).

#### Sequence accession numbers

The sequences were submitted to the Genbank dbEST database using the ESTin program [101] and are available publicly with the accession numbers FL634956-FL640828 (host gut tissue library) and FL641015-FL645753 (hindgut symbiont library). Annotated accession numbers are provided in Additional files 3, 4, 7 and 10.

## Competing interests

Pending US patent 61/168,275.

## Authors' contributions

DGB and MES designed the research; AT, MMW, MRC, DGB and MES performed the research; AT, DGB and MES analyzed the data;AT, DGB and MES wrote the paper; XZ provided technical assistance.

## Supplementary Material

Additional file 1 **Table S1 - Carbohydrate active genes, gut (host) library**. Summary of glycoside hydrolase (GH), glycosyl transferase (GT), carbohydrate esterase (CE), carbohydrate binding modules (CBM) and other miscellaneous (Misc.) carbohydrate active domain protein coding genes identified from the termite gut (host) library sequencing. Accession Numbers are provided in Additional file 3. Click here for file

Additional file 2 **Table S2 - Carbohydrate active genes, SYMBIONT library**. Summary of glycoside hydrolase (GH), glycosyl transferase (GT), carbohydrate esterase (CE), carbohydrate binding modules (CBM) and other miscellaneous (Misc.) carbohydrate active domain protein coding genes identified from the symbiont library sequencing. Accession Numbers are provided in Additional file 4. Click here for file

Additional file 3 **Table S3**. Genbank accession numbers for carbohydrate active enzymes, gut (host) library. Click here for file

Additional file 4 **Table S4**. Genbank accession numbers for carbohydrate active enzymes, SYMBIONT library. Click here for file

Additional file 5 **Table S5 - Dockerin, Fe-hydrogenase, ferredoxin oxidoreductase and nitroreductase genes, SYMBIONT library**. Summary of dockerin, Fe-hydrogenase, ferredoxin oxidoreductase and nitroreductase genes identified from the symbiont library sequencing. Accession Numbers are provided in Additional file 10. Click here for file

Additional file 6 **Table S6 - Candidate lignase, detoxification and antioxidant genes, gut (host) library**. Summary of candidate lignin degradation, detoxification and antioxidant enzyme coding genes identified from the termite gut (host) library sequencing. Accession Numbers are provided in Additional file 7. Click here for file

Additional file 7 **Table S7**. Genbank accession Nos. for candidate lignase, detoxification and antioxidant genes, gut (HOST) library. Click here for file

Additional file 8 **Figure S1**. Deduced amino acid alignment of the *R. flavipes *laccase contig obtained in the present study (Contig 659; indicated by arrows) with homologous insect and fungal laccases. The fungal laccases shown play known roles in lignin degradation. Shaded amino acids are those that match the *R. flavipes *sequence; insect sequences are above the arrows and fungal sequences are below. The ESTs assembling into the *R. flavipes *contig are as follows: FL639514, FL640712, FL635040, FL635071, FL635132, and FL635524. Sequence accession numbers for all homologs are shown in parentheses: Ms (*Manduca sexta*), Tribolium (*T. castaneum*), Ag (*Anopheles gambiae*), Termitomyces (*Termitomyces *sp. NS/Mg.), C. cinera (*Coprinus cinereus*), A. bisporus (*Agaricus bisporus*). Click here for file

Additional file 9 **Table S8**. PCR primer sequences used for validating laccase and catalase gene expression relative to the control gene β-actin. Click here for file

Additional file 10 **Table S9**. Genbank accession Nos. for Dockerin, Fe-hydrogenase, ferredoxin oxidoreductase and nitroreductase genes, SYMBIONT library. Click here for file
