# Supplementary material for: Parallel metatranscriptome analyses of host and symbiont gene expression in the gut of the termite Reticulitermes flavipes
Source: Biotechnol Biofuels. 2009 Oct 15;2:25. doi: 10.1186/1754-6834-2-25 (PMC2768689; doi:10.1186/1754-6834-2-25)
Supplement: Additional file 1 — Table S1 - Carbohydrate active genes, gut (host) library. Summary of glycoside hydrolase (GH), glycosyl transferase (GT), carbohydrate esterase (CE), carbohydrate binding modules (CBM) and other miscellaneous (Misc.) carbohydrate active domain protein coding genes identified from the termite gut (host) library sequencing. Accession Numbers are provided in Additional file 3. [file 1754-6834-2-25-S1.DOC]

**Table S1. Carbohydrate active genes, gut (host) library.**

Table S1. Continued.

Table S1. Continued.

Table S1. Continued.

Table S1. Continued.
