# Supplementary material for: Parallel metatranscriptome analyses of host and symbiont gene expression in the gut of the termite Reticulitermes flavipes
Source: Biotechnol Biofuels. 2009 Oct 15;2:25. doi: 10.1186/1754-6834-2-25 (PMC2768689; doi:10.1186/1754-6834-2-25)
Supplement: Additional file 2 — Table S2 - Carbohydrate active genes, SYMBIONT library. Summary of glycoside hydrolase (GH), glycosyl transferase (GT), carbohydrate esterase (CE), carbohydrate binding modules (CBM) and other miscellaneous (Misc.) carbohydrate active domain protein coding genes identified from the symbiont library sequencing. Accession Numbers are provided in Additional file 4. [file 1754-6834-2-25-S2.DOC]

**Table S2. Carbohydrate active genes, SYMBIONT library.**

Table S2. Continued,

Table S2. Continued,

Table S2. Continued,
