# Supplementary material for: Parallel metatranscriptome analyses of host and symbiont gene expression in the gut of the termite Reticulitermes flavipes
Source: Biotechnol Biofuels. 2009 Oct 15;2:25. doi: 10.1186/1754-6834-2-25 (PMC2768689; doi:10.1186/1754-6834-2-25)
Supplement: Additional file 3 — Table S3. Genbank accession numbers for carbohydrate active enzymes, gut (host) library. [file 1754-6834-2-25-S3.DOC]

**Table S3**. Genbank accession numbers for carbohydrate active enzymes, gut (host) library.

| **CAZY** | **EST ID** | **Genbank Nos.** |
| --- | --- | --- |
|  |  |  |
| GH1 | TG_309_K21 | FL635576 |
| GH1 (+GH5) | TG_Contig 771 | FL640173 FL639268 FL637619 FL637754 FL639498 FL636506 FL636125 FL635251 |
|  |  |  |
| GH2 | TG_303_L4 | FL636585 |
|  |  |  |
| GH3 | TG_08_C12 | FL637959 |
| GH3 | TG_15_A4 | FL638658 |
|  |  |  |
| GH5 (+GH1) | TG_Contig 771 | FL640173 FL639268 FL637619 FL637754 FL639498 FL636506 FL636125 FL635251 |
| GH5 | TG_28_G2 | FL640070 |
| GH5 | TG_31_B8 | FL640097 |
|  |  |  |
| GH7 | TG_Contig 113 | FL637680 FL635833 |
| GH7 | TG_Contig 167 | FL636267 FL637573 |
| GH7 | TG_09_D5 | FL638321 |
| GH7 | TG_10_D12 | FL638057 |
| GH7 | TG_22_C8 | FL639436 |
| GH7 | TG_23_H10 | FL639317 |
| GH7 | TG_30_E7 | FL640230 |
| GH7 | TG_306_M10 | FL637252 |
| GH7 | TG_310_E8 | FL635693 |
| GH7 | TG_37_A6 | FL640739 |
|  |  |  |
| GH8 | TG_11_A4 | FL638145 |
|  |  |  |
| GH9 (*Cell-1*) | TG_Contig 630 | FL639343 FL638695 FL639554 FL637616 FL640495 FL639184 FL639540 CB518295 FL636656 CB518296 FL635731 |
| GH9 | TG_14_B9 | FL638597 |
|  |  |  |
| GH10 (+GH53) | TG_Contig 141 | FL637953 FL644050 FL636080 |
|  |  |  |
| GH11 | TG_Contig 184 | FL638953 FL636366 FL643477 FL641536 FL644766 FL642851 FL644625 FL644716 FL643102 FL645522 |
| GH11 | TG_Contig 613 | FL639270 FL640273 FL638067 FL635042 |
| GH11 | TG_21_D5 | FL639281 |
| GH11 | TG_23_E2 | FL639373 |
| GH11 | TG_309_J21 | FL636065 |
|  |  |  |
| GH13 | TG_Contig 244 | FL638647 FL637787 FL636738 |
| GH13 | TG_Contig 660 | FL639510 FL639382 FL636180 FL637565 |
| GH13 | TG_02_C3 | FL637565 |
| GH13 | TG_04_D4 | FL637758 |
| GH13 | TG_11_F12 | FL638109 |
| GH13 | TG_14_E2 | FL638619 |
| GH13 | TG_15_G5 | FL638734 |
| GH13 | TG_23_E6 | FL639382 |
| GH13 | TG_Contig 650 | FL638753 FL639460 FL637612 |
| GH13 (+GH70) | TG_Contig 541 | FL638881 FL635848 |
|  |  |  |
| GH16 | TG_Contig 702 | FL638539 FL638865 FL637570 FL640180 FL640009 FL639757 FL640074 FL636329 FL635911 FL636763 |
| GH16 | TG_Contig 610 | FL639260 FL635644 FL636862 |
| GH16 | TG_302_K4 | FL636194 |
|  |  |  |
| GH18 | TG_Contig 339 | FL637567 FL640002 FL638578 FL640672 FL636033 |
| GH18 | TG_Contig 532 | FL638804 FL636448 |
| GH18 | TG_Contig 726 | FL640357 FL638483 FL640713 FL639415 FL638081 FL636234  FL639879 FL638090 FL635238 FL639902 FL637343 FL637212 FL637298 |
| GH18 | TG_01_D11 | FL639799 |
| GH18 | TG_14_B7 | FL638578 |
| GH18 | TG_29_C1 | FL639928 |
| GH18 | TG_302_O13 | FL636033 |
| GH18 (+CBM14) | TG_Contig 322 | FL637931 FL637765 FL637422 FL641416 |
|  |  |  |
| GH20 | TG_Contig 332 | FL638685 FL637515 FL640518 FL636046 FL637491 FL636660 FL636621 FL636318 |
| GH20 | TG_Contig 670 | FL640152 FL639569 FL636522 |
| GH20 | TG_303_E21 | FL636318 |
| GH20 | TG_309_M8 | FL635496 |
|  |  |  |
| GH26 | TG_305_A12 | FL637053 |
|  |  |  |
| GH27 | TG_Contig 559 | FL638987 FL635520 |
|  |  |  |
| GH30 | TG_Contig 643 | FL639424 FL637190 |
| GH30 | TG_25_B12 | FL639620 |
| GH30 | TG_301_E21 | FL635079 |
|  |  |  |
| GH37 | TG_29_F7 | FL639972 |
|  |  |  |
| GH38 | TG_18_E6 | FL639002 |
| GH38 | TG_301_O23 | FL635063 |
|  |  |  |
| GH43 | TG_304_K16 | FL636820 |
|  |  |  |
| GH45 | TG_Contig 118 | FL640466 FL638098 FL643415 FL644599 FL635101 FL635859 |
|  |  |  |
| GH53 (+GH10) | TG_Contig 141 | FL637953 FL644050 FL636080 |
|  |  |  |
| GH76 | TG_18_H5 | FL639033 |
|  |  |  |
| GH85 | TG_34_C9 | FL640489 |
|  |  |  |
| GT1 | TG_Contig 646 | FL639447 FL636477 FL636602 |
| GT1 | TG_Contig 705 | FL639767 FL637181 |
| GT1 | TG_Contig 807 | FL640385 FL636100 FL635450 FL639977 FL635408 |
| GT1 | TG_11_A7 | FL638141 |
| GT1 (*) | TG_304_F6 | FL636860 |
| GT1 (+GT28) | TG_Contig 194 | FL636418 FL635094 |
| GT1 (+GT28) | TG_305_L9 | FL637012 |
|  |  |  |
| GT2 | TG_Contig 214 | FL636541 FL637150 |
|  |  |  |
| GT3 | TG_Contig 835 | FL640572 FL635779 |
|  |  |  |
| GT4 | TG_03_H1 | FL637707 |
| GT4 | TG_309_M23 | FL635567 |
|  |  |  |
| GT10 | TG_Contig 665 | FL639543 FL635024 FL639348 FL637265 FL639628 FL636083 FL636724 |
| GT10 | TG_25_C8 | FL639628 |
| GT10 | TG_302_B19 | FL636083 |
| GT10 | TG_304_D23 | FL636726 |
|  |  |  |
| GT13 | TG_305_L3 | FL637013 |
|  |  |  |
| GT22 | TG_30_G12 | FL640261 |
|  |  |  |
| GT28 | TG_Contig 439 | FL638255 FL639778 FL639945 FL640004 FL637804 FL637036 FL635359 |
| GT28 (+GT1) | TG_Contig 194 | FL636418 FL635094 |
| GT28 (+GT1) | TG_305_L9 | FL637012 |
|  |  |  |
| GT66 | TG_Contig 527 | FL638905 FL638780 FL637714 FL638375 FL634980 FL637775 FL638045 FL636832 |
| GT66 | TG_302_B11 | FL636084 |
| GT66 | TG_33_H8 | FL640364 |
|  |  |  |
| N/A | TG_23_F5 | FL639368 |
|  |  |  |
| CE4 | TG_Contig 313 | FL636264 FL637347 FL636937 |
| CE4 | TG_Contig 612 | FL639269 FL640800 FL635638 |
| CE4 | TG_309_G23 | FL635548 |
|  |  |  |
| N/A | TG_Contig 282 | FL634976 FL637502 FL637021 FL644380 FL638705 FL638204 FL638160 FL641636 |
| N/A | TG_Contig 425 | FL638160 FL638204 FL636193 FL638705 FL644380 FL637028 FL641636 |
| N/A | TG_305_A1 | FL637054 |
|  |  |  |
| CBM13 | TG_Contig 349 | FL638897 FL639074 FL643277 FL639461 FL643149 FL640818 FL635826 FL638941 FL637635 FL636014 |
| CBM13 | TG_Contig 553 | FL640818 FL638941 FL643277 FL639074 FL638897 FL639461 FL643149 FL635826 FL637635 FL636014 FL637282 FL635910 |
| CBM13 | TG_304_A17 | FL636615 |
|  |  |  |
| CBM14 | TG_Contig 166 | FL639577 FL639006 FL640588 FL640551 FL639775 FL636263 FL636405 FL636251 |
| CBM14 | TG_Contig 190 | FL635012 FL636384 FL636190 |
| CBM14 | TG_Contig 473 | FL638494 FL637824 FL638311 FL637330 FL636440 FL637273 FL636362 |
| CBM14 | TG_Contig 519 | FL639103 FL640601 FL640745 FL638723 FL640374 FL639385 FL636723 FL637641 FL637639 FL641696 BJ979301 |
| CBM14 | TG_Contig 557 | FL639127 FL638031 FL635017 FL638284 FL639994 FL638025 FL638960 FL638118 FL636529 FL637043 FL637104 |
| CBM14 | TG_Contig 718 | FL639828 FL636429 |
| CBM14 | TG_23_D10 | FL639385 |
| CBM14 | TG_29_G4 | FL639980 |
| CBM14 | TG_305_G6 | FL637077 |
| CBM14 | TG_309_E23 | FL635547 |
| CBM14 | TG_32_B12 | FL640293 |
| CBM14 (+GH18) | TG_Contig 322 | FL637931 FL637765 FL637422 FL641416 |
|  |  |  |
| CBM33 | TG_Contig 314 | FL637350 FL636407 |
| CBM33 | TG_20_F8 | FL639201 |
|  |  |  |
| N/A | TG_Contig 427 | FL638172 FL639244 FL640748 FL637853 FL637770 |
| N/A | TG_Contig 649 | FL640508 FL637524 |
|  |  |  |
| Misc. | TG_00_D3 | FL634975 |
|  |  |  |
| Misc. | TG_Contig 153 | FL640558 FL640328 FL639501 FL639495 FL636163 FL637731 FL636236 |
| Misc. | TG_Contig 228 | FL636471 FL636625 |
| Misc. | TG_Contig 256 | FL636823 FL636531 |
| Misc. | TG_Contig 388 | FL639618 FL638297 FL637556 FL638488 FL639583 |
| Misc. | TG_Contig 501 | FL638623 FL638915 FL637798 |
| Misc. | TG_Contig 579 | FL639099 FL636027 |
| Misc. | TG_Contig 787 | FL640319 FL637076 |
| Misc. | TG_07_C5 | FL637844 |
| Misc. | TG_12_G12 | FL638464 |
| Misc. | TG_23_G4 | FL639397 |
| Misc. | TG_26_B11 | FL639718 |
| Misc. | TG_304_N4 | FL636883 |
| Misc. | TG_305_C15 | FL636921 |
| Misc. | TG_309_C17 | FL635535 |
| Misc. | TG_31_C1 | FL640110 |
| Misc. | TG_310_B21 | FL635595 |
| Misc. | TG_23_H3 | FL639404 |
