# Supplementary material for: Parallel metatranscriptome analyses of host and symbiont gene expression in the gut of the termite Reticulitermes flavipes
Source: Biotechnol Biofuels. 2009 Oct 15;2:25. doi: 10.1186/1754-6834-2-25 (PMC2768689; doi:10.1186/1754-6834-2-25)
Supplement: Additional file 4 — Table S4. Genbank accession numbers for carbohydrate active enzymes, SYMBIONT library. [file 1754-6834-2-25-S4.DOC]

**Table S4. Genbank accession numbers for carbohydrate active enzymes, SYMBIONT library.**

| **CAZY** | **EST ID** | **Genbank Nos.** |
| --- | --- | --- |
|  |  |  |
| GH2 | TS54-H2 | FL645624 |
|  |  |  |
| GH3 | TS05-H1 | FL641548 |
| GH3 | TS36-B4 | FL644155 |
| GH3 | TS51-B11 | FL645414 |
| GH3 | TS17-C6 | FL642454 |
| GH3 | TS22-D11 | FL642936 |
| GH3 | TS10-H10 | FL641822 |
| GH3 | TS19-F12 | FL642593 |
| GH3 | TS26-E5 | FL643199 |
| GH3 | TS50-B7 | FL645723 |
| GH3 | TS-contig 278 | FL644791 FL641703 FL645694 |
|  |  |  |
| GH5 | TS-contig 106 | FL643408 FL643633 FL645194 FL643522 FL642133 FL641469 FL643252 FL642619 FL642794 FL643521 FL640070  FL644362 FL643394 FL644399 FL645086 |
| GH5 | TS-contig 243 | FL641469 FL643252 FL642794 FL644362 FL643408 FL643633 FL642133 FL644399 FL643521 FL640070 FL643522  FL642619 FL645194 |
| GH5 | TS05-C11 | FL641498 |
| GH5 | TS08-F6 | FL641604 |
| GH5 | TS13-C8 | FL642108 |
| GH5 | TS20-C1 | FL642731 |
| GH5 | TS22-E4 | FL642939 |
| GH5 | TS27-D6 | FL643291 |
| GH5 | TS47-D7 | FL645086 |
| GH5 | TS47-H5 | FL645129 |
| GH5 | TS55-H11 | FL645629 |
|  |  |  |
| GH7 | TS-contig 41 | FL645581 FL645594 FL645587 |
| GH7 | TS-contig 56 | FL645708 FL643260 |
| GH7 | TS-contig 70 | FL644726 FL642558 FL644837 FL643410 FL643323 FL644076 FL642305 FL642701 FL644572 FL645546 FL645342  FL644173 FL644988 FL642821 FL643822 FL645700 FL645698 FL643688 FL644998 FL644795 FL644891 FL644248  FL642753 FL643662 FL643561 FL642282 FL639317 FL644032 FL644843 FL644540 FL644502 FL642674 FL643664  FL644483 |
| GH7 | TS-contig 95 | FL644101 FL642538 |
| GH7 | TS-contig 111 | FL642655 FL644945 FL645750 FL641078 FL642196 FL643062 FL642742 FL642924 FL643913 FL643945 FL641643  FL644327 FL643885 FL644351 FL642144 FL641658 FL642340 FL645287 |
| GH7 | TS-contig 121 | FL642008 FL642769 |
| GH7 | TS-contig 128 | FL642828 FL642229 |
| GH7 | TS-contig 141 | FL642987 FL643423 |
| GH7 | TS-contig 195 | FL643662 FL642282 FL644248 FL644891 FL643664 FL644032 FL643118 FL639317 FL644726 FL642558 FL643410  FL643030 FL644173 FL641301 FL645599 FL644837 FL645042 FL644572 FL642305 FL643236 FL642146 FL645546  FL642049 FL642701 FL645698 |
| GH7 | TS-contig 206 | FL645365 FL645187 FL643682 FL645680 FL643957 FL644627 FL642181 FL644227 FL643105 FL644603 FL644988  FL641239 FL644726 FL643822 FL642558 FL644998 FL643688 FL645700 FL643030 FL645698 FL636267 FL644837  FL643410 FL644795 FL644572 FL645599 FL644173 FL642305 FL642674 FL645042 FL643236 FL642753 FL644483  FL644952 FL643561 FL645689 FL642049 FL645546 FL644540 FL642701 FL645046 FL642097 FL644843 FL644018  FL643194 FL642146 FL644413 |
| GH7 | TS-contig 222 | FL641964 FL644843 FL644173 FL643561 FL644952 FL643030 FL645599 FL642055 FL642049 FL645042 FL643236  FL645689 FL641301 FL643194 FL642097 FL644502 FL644726 FL641912 FL642558 FL643410 FL644572 FL644837  FL642305 FL644891 FL645698 FL645700 FL644988 FL645546 FL644032 FL644795 FL644998 FL643822 FL643688  FL643662 FL642282 FL644248 FL641785 FL643664 FL642753 FL639317 DC238592 FL645187 FL645365 |
| GH7 | TS-contig 225 | FL644253 FL643089 FL638321 |
| GH7 | TS-contig 256 | FL645700 FL645698 FL644988 FL642753 FL641758 FL644795 FL643479 FL642674 FL643822 FL644998 FL643688  FL644018 FL644483 FL644540 FL645046 FL641203 FL644726 FL642558 FL643030 FL644413 FL643410 FL645599  FL643236 FL645042 FL645365 FL644572 FL645187 FL641301 FL644952 FL642305 FL644173 FL645546 FL644891  FL642049 FL645689 FL643561 FL644859 FL642097 FL643194 FL645525 FL643105 FL643664 FL644843 FL644502 |
| GH7 | TS-contig 283 | FL644891 FL643664 FL644248 FL639317 FL643662 FL642282 FL642146 FL643118 FL643702 FL644726 FL642558  FL644952 FL643030 FL643561 FL644837 FL642281 FL645599 FL644173 FL645042 FL642049 FL642753 FL644032  FL643410 FL644988 FL645689 FL642097 FL645700 FL644843 FL641301 FL645545 FL642701 FL643688 FL642674  FL645698 FL644572 FL642305 FL644483 FL645546 FL644018 FL644795 |
| GH7 | TS-contig 295 | FL643822 FL642753 FL643688 FL644998 FL642674 FL644988 FL644018 FL645046 FL645700 FL644483 FL641203  FL645698 FL643479 FL644795 FL644859 FL644540 FL644726 FL642558 FL641758 FL643030 FL643410 FL645599  FL644837 FL645365 FL645042 FL644413 FL645187 FL643236 FL641301 FL644173 FL644572 FL644952 FL645525  FL642305 FL645546 FL641158 FL642049 FL642701 |
| GH7 | TS-contig 321 | FL645254 FL644575 |
| GH7 | TS-contig 326 | FL645416 FL642890 FL642393 FL645327 |
| GH7 | TS-contig 343 | FL644952 FL643030 FL645599 FL645042 FL641912 FL643236 FL642049 FL645689 FL643194 FL642097 FL643561  FL644173 FL644843 FL641785 FL641301 FL641964 FL644502 FL642055 FL645700 FL645698 FL643822 FL644988  FL644795 FL644032 FL643688 FL644998 FL642753 FL645187 FL644248 FL645365 FL643662 FL642282 FL644540  FL645046 FL639317 FL642674 FL644483 |
| GH7 | TS03-G3 | FL641355 |
| GH7 | TS04-A3 | FL641381 |
| GH7 | TS09-F4 | FL641672 |
| GH7 | TS11-G5 | FL641958 |
| GH7 | TS15-A7 | FL642345 |
| GH7 | TS15-C12 | FL642371 |
| GH7 | TS22-A2 | FL642896 |
| GH7 | TS25-D9 | FL643107 |
| GH7 | TS28-E2 | FL643378 |
| GH7 | TS31-A1 | FL643682 |
| GH7 | TS31-H1 | FL643759 |
| GH7 | TS39-H1 | FL644686 |
| GH7 | TS43-H1 | FL644846 |
| GH7 | TS43-H7 | FL644859 |
| GH7 | TS45-G11 | FL644672 |
| GH7 | TS52-D4 | FL645353 |
| GH7 | TS53-G7 | FL645542 |
|  |  |  |
| GH8 | TS-contig 228 | FL643816 FL644267 FL642455 |
|  |  |  |
| GH10 | TS-contig 301 | FL644914 FL645095 |
| GH10 (+GH53) | TS35-A12 | FL644050 |
|  |  |  |
| GH11 | TS-contig 15 | DN792510 FL645433 DN792496 FL645118 FL645372 FL644193 FL645274 FL640292 FL645204 FL644596 FL638680  FL641870 FL641165 BQ788160 FL645692 FL638348 FL641761 FL635762 FL637866 FL642217 FL641409 FL639773  FL636488 FL641442 CB518306 FL644927 ES597834 FL641503 FL635285 FL636676 |
| GH11 | TS-contig 33 | FL644858 FL645358 |
| GH11 | TS-contig 262 | FL644625 FL644617 |
| GH11 | TS-contig 263 | FL645488 FL644645 |
| GH11 | TS23-D10 | FL642851 |
| GH11 | TS24-F6 | FL643477 |
| GH11 | TS39-D2 | FL644716 |
| GH11 | TS43-C2 | FL644790 |
| GH11 | TS49-G10 | FL645311 |
| GH11 | TS53-E12 | FL645522 |
|  |  |  |
| GH16 | TS00-F1 | FL641082 |
| GH16 | TS14-A12 | FL642176 |
| GH16 | TS22-H1 | FL642967 |
|  |  |  |
| GH18 | TS-contig 118 | FL643820 FL642723 |
| GH18 | TS06-D8 | FL641790 |
| GH18 | TS17-B4 | FL642439 |
| GH18 | TS38-H6 | FL644395 |
| GH18 | TS43-A6 | FL644773 |
|  |  |  |
| GH20 | TS42-C3 | FL644427 |
| GH20 | TS-contig 330 | FL644858 FL645358 |
| GH20 | TS48-E11 | FL645188 |
|  |  |  |
| GH26 | TS-contig 192 | FL643622 FL644037 FL643775 FL645610 FL637053 |
| GH26 | TS24-C9 | FL643447 |
| GH26 | TS39-D4 | FL644713 |
| GH26 | TS52-B1 | FL645330 |
| GH26 | TS55-H4 | FL645703 |
| GH26 (+CBM6) | TS25-C3 | FL643094 |
|  |  |  |
| GH30 | TS-contig 99 | FL642562 FL642563 |
|  |  |  |
| GH42 | TS29-A11 | FL643514 |
|  |  |  |
| GH45 | TS-contig 28 | FL643161 FL645349 FL642729 FL643009 |
| GH45 | TS-contig 325 | FL645307 FL643541 FL643415 FL642182 FL644599 |
| GH45 | TS50-G9 | FL645753 |
| GH45 | TS53-G4 | FL645536 |
|  |  |  |
| GH47 | TS08-E1 | FL641588 |
|  |  |  |
| GH53 (+GH10) | TS35-A12 | FL644050 |
|  |  |  |
| GH77 | TS16-H8 | FL642333 |
|  |  |  |
| GH92 | TS04-E4 | FL641428 |
|  |  |  |
|  |  |  |
| GT2 | TS04-B6 | FL641395 |
| GT2 | TS22-C5 | FL642920 |
|  |  |  |
| GT8 | TS-contig 13 | FL644764 FL641146 |
| GT8 | TS-contig 167 | FL643308 FL643977 |
| GT8 | TS25-D1 | FL643100 |
| GT8 | TS37-H2 | FL644305 |
|  |  |  |
|  |  |  |
| CE4 | TS-contig 252 | FL644020 FL644497 |
| CE4 | TS48-H11 | FL645136 |
| N/A | TS-contig 173 | FL644380 FL641636 FL643381 FL638705 FL638160 FL638204 FL636193 |
|  |  |  |
|  |  |  |
| CBM6 | TS11-A6 | FL641905 |
| CBM6 (+GH26) | TS25-C3 | FL643094 |
|  |  |  |
| CBM13 | TS-contig 156 | FL643149 FL643277 FL638897 FL639074 FL639461 FL640818 FL638941 FL635826 FL637635 FL636014 |
| CBM13 | TS22-H5 | FL642886 |
|  |  |  |
| CBM20 | TS39-A11 | FL644691 |
|  |  |  |
|  |  |  |
| Misc. | TS-contig 251 | FL641345 FL644495 FL641329 FL640782 |
| Misc. | TS03-G12 | FL641361 |
| Misc. | TS16-F4 | FL642307 |
| Misc. | TS39-G8 | FL644758 |
|  |  |  |
| Misc. | TS-contig 18 | FL644528 FL641204 |
| Misc. | TS00-E6 | FL641076 |
| Misc. | TS13-A11 | FL642086 |
| Misc. | TS45-E7 | FL644641 |
| Misc. | TS47-D9 | FL645093 |
|  |  |  |
