# Supplementary material for: Parallel metatranscriptome analyses of host and symbiont gene expression in the gut of the termite Reticulitermes flavipes
Source: Biotechnol Biofuels. 2009 Oct 15;2:25. doi: 10.1186/1754-6834-2-25 (PMC2768689; doi:10.1186/1754-6834-2-25)
Supplement: Additional file 5 — Table S5 - Dockerin, Fe-hydrogenase, ferredoxin oxidoreductase and nitroreductase genes, SYMBIONT library. Summary of dockerin, Fe-hydrogenase, ferredoxin oxidoreductase and nitroreductase genes identified from the symbiont library sequencing. Accession Numbers are provided in Additional file 10. [file 1754-6834-2-25-S5.DOC]

**Table S5. Dockerin, Fe-hydrogenase, ferredoxin oxidoreductase and nitroreductase genes, SYMBIONT library.**
