# Supplementary material for: Parallel metatranscriptome analyses of host and symbiont gene expression in the gut of the termite Reticulitermes flavipes
Source: Biotechnol Biofuels. 2009 Oct 15;2:25. doi: 10.1186/1754-6834-2-25 (PMC2768689; doi:10.1186/1754-6834-2-25)
Supplement: Additional file 6 — Table S6 - Candidate lignase, detoxification and antioxidant genes, gut (host) library. Summary of candidate lignin degradation, detoxification and antioxidant enzyme coding genes identified from the termite gut (host) library sequencing. Accession Numbers are provided in Additional file 7. [file 1754-6834-2-25-S6.DOC]

**Table S6. Candidate lignase, detoxification and antioxidant genes, gut (host) library.**
