# Supplementary material for: Parallel metatranscriptome analyses of host and symbiont gene expression in the gut of the termite Reticulitermes flavipes
Source: Biotechnol Biofuels. 2009 Oct 15;2:25. doi: 10.1186/1754-6834-2-25 (PMC2768689; doi:10.1186/1754-6834-2-25)
Supplement: Additional file 7 — Table S7. Genbank accession Nos. for candidate lignase, detoxification and antioxidant genes, gut (HOST) library. [file 1754-6834-2-25-S7.DOC]

**Table S7. Genbank accession Nos. for candidate lignase, detoxification and antioxidant genes, gut (HOST) library.**

| **Family** | **EST ID** | **Genbank Nos.** |
| --- | --- | --- |
|  |  |  |
| Laccase | TG_Contig 659 | FL639504 FL640712 FL635040 FL635071 FL635132 FL635524 |
|  |  |  |
| Catalase | TG_Contig 230 | FL640175 FL639709 FL640611 FL640731 FL638122 FL638283 FL635175 FL636629 |
|  |  |  |
| Epoxide Hydrolase | TG_Contig 840 | FL640608 FL636393 FL638670 FL635113 |
|  | TG_15_A8 | FL638670 |
|  |  |  |
| Superoxide Disumtase | TG_Contig 272 | FL636626 FL636951 FL636064 |
|  | TG_Contig 463 | FL638431 FL635991 FL637066 FL637255 |
|  | TG_Contig 640 | FL639405 FL636654 |
|  |  |  |
| Glutathione Peroxidase | TG_Contig 57 | FL637958 FL637623 FL639779 FL638608 FL635382 FL635424 FL635921 |
|  | TG_Contig 209 | FL636507 FL636578 FL635370 FL635369 |
|  | TG_Contig 413 | FL638693 FL637601 FL638056 FL637645 |
|  | TG_Contig 811 | FL639189 FL640407 FL638203 FL640403 FL638076 FL640506 FL637342 FL638175 |
|  | TG_14_D2 | FL638608 |
|  | TG_310_O15 | FL635921 |
|  |  |  |
| Glutathione-S-transferase | TG_Contig 3 | FL638367 FL640297 FL639093 FL634986 FL636346 FL640045 FL635047 FL637027 FL636272 FL635291 |
|  | TG_Contig 116 | FL640327 FL638845 FL640585 FL638197 FL635847 FL636395 |
|  | TG_Contig 215 | FL638625 FL636543 FL637867 FL636855 FL637232 FL636804 |
|  | TG_Contig 318 | FL638233 FL640112 FL639973 FL635422 FL637404 FL635290 FL636756 FL635181 FL637433 FL636785 |
|  | TG_Contig 812 | FL638024 FL639682 FL640600 FL640414 FL635303 FL638381 FL636971 FL635373 FL636284 FL636013 FL636035 FL635339 |
|  | TG_301_E17 | FL635080 |
|  |  |  |
| Alcohol Dehydrogenase | TG_Contig 16 | FL639267 FL638035 FL640471 FL635070 |
|  | TG_Contig 199 | FL634972 FL636451 FL640734 FL636884 FL635081 FL635212 |
|  | TG_Contig 331 | FL638305 FL637513 |
|  | TG_Contig 397 | FL638731 FL639652 FL637951 FL635349 FL635942 FL639810 FL636050 |
|  | TG_Contig 506 | FL638651 FL638503 |
|  | TG_Contig 839 | FL638327 FL638264 FL639188 FL640605 FL638363 |
|  | TG_25_H12 | FL639607 |
|  |  |  |
| Carboxylesterase | TG_Contig 275 | FL638979 FL640151 FL636973 |
|  | TG_Contig 493 | FL638587 FL637361 FL637132 FL636639 FL638245 |
|  | TG_Contig 515 | FL638851 FL638692 FL637886 FL638877 FL635365 |
|  | TG_11_C1 | FL638162 |
|  | TG_15_C3 | FL638686 |
|  | TG_17_B6 | FL638877 |
|  | TG_26_A12 | FL639693 |
|  | TG_306_D9 | FL637274 |
|  | TG_34_D6 | FL640498 |
|  | TG_36_A5 | FL640662 |
|  | TG_306_K13 | FL637352 |
|  | TG_31_H10 | FL640085 |
|  | TG_310_H12 | FL635797 |
|  |  |  |
| Cytochrome P450 | TG_Contig 357 | FL639428 FL635872 BQ788187 FL637685 |
|  | TG_Contig 543 | FL640637 FL638893 FL640773 FL636262 FL635527 FL636088 FL636256 |
|  | TG_Contig 560 | FL638992 FL640597 FL636095 |
|  | TG_Contig 850 | FL638880 FL640694 FL639091 |
|  | TG_02_H8 | FL637535 |
|  | TG_07_B9 | FL637840 |
|  | TG_14_C1 | FL638592 |
|  | TG_14_C6 | FL638604 |
|  | TG_16_H8 | FL638762 |
|  | TG_20_B8 | FL639157 |
|  | TG_23_B10 | FL639337 |
|  | TG_29_F10 | FL639974 |
|  | TG_306_M3 | FL637360 |
|  | TG_310_F21 | FL635626 |
|  | TG_310_P20 | FL635753 |
|  | TG_37_B8 | FL640754 |
