# Supplementary material for: Parallel metatranscriptome analyses of host and symbiont gene expression in the gut of the termite Reticulitermes flavipes
Source: Biotechnol Biofuels. 2009 Oct 15;2:25. doi: 10.1186/1754-6834-2-25 (PMC2768689; doi:10.1186/1754-6834-2-25)
Supplement: Additional file 8 — Figure S1. Deduced amino acid alignment of the R. flavipes laccase contig obtained in the present study (Contig 659; indicated by arrows) with homologous insect and fungal laccases. The fungal laccases shown play known roles in lignin degradation. Shaded amino acids are those that match the R. flavipes sequence; insect sequences are above the arrows and fungal sequences are below. The ESTs assembling into the R. flavipes contig are as follows: FL639514, FL640712, FL635040, FL635071, FL635132, and FL635524. Sequence accession numbers for all homologs are shown in parentheses: Ms (Manduca sexta), Tribolium (T. castaneum), Ag (Anopheles gambiae), Termitomyces (Termitomyces sp. NS/Mg.), C. cinera (Coprinus cinereus), A. bisporus (Agaricus bisporus). [file 1754-6834-2-25-S8.DOC]

**Figure S1.**

Figure S1, continued.
