# Supplementary material for: Parallel metatranscriptome analyses of host and symbiont gene expression in the gut of the termite Reticulitermes flavipes
Source: Biotechnol Biofuels. 2009 Oct 15;2:25. doi: 10.1186/1754-6834-2-25 (PMC2768689; doi:10.1186/1754-6834-2-25)
Supplement: Additional file 9 — Table S8. PCR primer sequences used for validating laccase and catalase gene expression relative to the control gene β-actin. [file 1754-6834-2-25-S9.DOC]

**Table S8.** qPCR primer sequences.

| **Primer Name** | **Alternate Name** | **Sequence (5’ – 3’)** |
| --- | --- | --- |
| Laccase 1L | Contig659-1R | CCCGGACATCAACTATCTTCTC |
| Laccase 1R | Contig659-1L | GCATAGGATGTCGTCTGGTACA |
| Laccase 2L | Contig659-2R | CTCCCGGACATCAACTATCTTC |
| Laccase 2R | Contig659-2L | GCATAGGATGTCGTCTGGTACA |
| Catalase 3L | Contig230-3R | GGATAACTTTAGCCAGCCCTCT |
| Catalase 3R | Contig230-3L | CGTTCCTGTACAAAGTCAGCAG |
| Catalase 4L | Contig230-4R | CTGCAGATTCCAGTGAACTGTC |
| Catalase 4R | Contig230-4L | CCTGTACAAAGTCAGCAGCATC |
| RfEst3 L | Contig275L-set5 | AGAGACGGTTGAAGACTTGGT |
| RfEst3 R | Contig275R-set5 | ACTGATACGTAGTGGCCTGACA |
| b-actin L | N/A | AGAGGGAAATCGTGCGTGAC |
| b-actin R | N/A | CAATAGTGATGACCTGGCCGT |
