# Supplementary material for: Parallel metatranscriptome analyses of host and symbiont gene expression in the gut of the termite Reticulitermes flavipes
Source: Biotechnol Biofuels. 2009 Oct 15;2:25. doi: 10.1186/1754-6834-2-25 (PMC2768689; doi:10.1186/1754-6834-2-25)
Supplement: Additional file 10 — Table S9. Genbank accession Nos. for Dockerin, Fe-hydrogenase, ferredoxin oxidoreductase and nitroreductase genes, SYMBIONT library. [file 1754-6834-2-25-S10.DOC]

**Table S9. Genbank accession Nos. for Dockerin, Fe-hydrogenase, ferredoxin oxidoreductase and nitroreductase genes, SYMBIONT library.**

|  | **CONTIG ID** | **Genbank Nos.** |  |  |
| --- | --- | --- | --- | --- |
|  |  |  |  |  |
| **Dockerin / Cellulosome** | | |  |  |
|  | TS33-G3 | FL643934 |  |  |
|  | TS25-B5 | FL643080 |  |  |
|  |  |  |  |  |
| **Fe-Hydrogenases** | |  |  |  |
|  | TS45-E3 | FL644639 |  |  |
|  | TS27-B12 | FL643270 |  |  |
|  | TS48-F2 | FL645197 |  |  |
|  | TS52-E2 | FL645360 |  |  |
|  | TS41-F4 | FL644558 |  |  |
|  |  |  |  |  |
| **Ferrodoxin-oxidoreductases** | | |  |  |
|  | TS46-F9 | FL645026 |  |  |
|  | TS39-G4 | FL644755 |  |  |
|  | TS43-E3 | FL644820 |  |  |
|  | TS04-H2 | FL641457 |  |  |
|  | TS14-B9 | FL642178 |  |  |
|  | TS25-E3 | FL643110 |  |  |
|  | TS28-C3 | FL643357 |  |  |
|  | TS44-E2 | FL644930 |  |  |
|  | TS53-E3 | FL645518 |  |  |
|  | TS03-B11 | FL641300 |  |  |
|  | TS-contig 223 | FL644174 FL641525 FL641089 | | |
|  | TS05-C1 | FL641491 |  |  |
|  |  |  |  |  |
| **Nitrogen reductase** | | |  |  |
|  | TS19-G8 | FL642600 |  |  |
|  | TS12-H2 | FL642058 |  |  |
|  | TS-contig 130 | FL642848 FL642039 | |  |
|  | TS14-G7 | FL642233 |  |  |
|  | TS52-G5 | FL645383 |  |  |
